# Phytochemicals and biological activities of *Ligularia* species

**DOI:** 10.1007/s13659-011-0003-y

**Published:** 2011-09-16

**Authors:** Jun-Li Yang, Rui Wang, Yan-Ping Shi

**Affiliations:** 1 State Key Laboratory of Applied Organic Chemistry, Lanzhou University, Lanzhou, 730000 China; 2 Key Laboratory of Chemistry of Northwestern Plant Resources and Key Laboratory for Natural Medicine of Gansu Province, Lanzhou Institute of Chemical Physics, Chinese Academy of Sciences, Lanzhou, 730000 China

## Abstract

*Ligularia*, an important genus of the Compositae family, has captured the interest of natural product chemists for years. Phytochemical investigations on the title genus have led to isolation of hundreds of secondary metabolites with various skeletons. Herein, we summarized the chemical constituents of this genus and their biological activities over the past few decades.

## Introduction

The genus *Ligularia* has been taxonomically placed in the Compositae (tribe Senecioneae) with more than 27 species used as folk remedies[1]. The systematic and in-depth phytochemical investigations on *Ligularia* species have resulted in hundreds of secondary metabolites with various skeletons and interesting biological activities have been discovered from this genus. The application of some *Ligularia* species in traditional medicines has been in period. For example, *L. sagitta* possess efficacies of relieving phlegm and cough, invigorating circulation of blood, soothing pain, and particularly curing rheumatoid arthritis[2]. *L. fischeri* has been used as a folk medicine for the treatment of coughs, inflammations, jaundice, scarlet fever, rheumatoidal arthritis, and hepatic diseases[3]. *L. veitchiana* was reported for the treatment of influenza, cough, ulcer and pulmonary tuberculosis[4]. *L. lapathifolia* has been used to treat cough and inflammation[5]. Furthermore, *L. sibirica* and *L. hodgsoni* are used as herbal remedies to treat bronchitis, cough, asthma, and phthisis[6].

Searching for bioactive molecules from nature source has always been our interest[7–12]. In the past years, some *Ligularia* species, such as *L. virgaurea* spp. *oligocephala*[9], *L. myriocephala*[13], and *L. fischeri*[14], have been investigated in our lab from the viewpoint of phytochemistry. The promising results stimulated our interest in *Ligularia* species as a source of substances with chemical and biological diversity. Here we review the state of the art in the phytochemical investigation and biological activity evaluation of *Ligularia* species in recent years (1990.1~2011.6).

## 1 Chemical Constituents

*To whom correspondence should be addressed. E-mail: shiyp@licp.cas.cn

**1.1 Sesquiterpenoids:** As the major chemical constituents, there are 289 sesquiterpenoids reviewed. These sesquiterpenoids comprise eremophilane-type (**1-1** to **1-210**), bisabolane-type (**1-211** to **1-242**), oplopane-type (**1-243** to **1-248**), guaiane and pseudoguaiane types (**1-249** to **1-253**), eudesmane type (**1-254** to **1-258**), and other skeleton types (**1-259** to **1-267**) as well as dimers (**1-268** to **1-289**). The names and corresponding plant sources of these sesquiterpenoids were listed in Table 1[1–6,9,13–99].

**1.1.1 Eremophilane Sesquiterpenoids:** Of the 368 secondary metabolites reviewed in this paper, there are 210 eremophilane sesquiterpenoids (**1-1** to **1-210**). Consequently, the eremophilane sesquiterpenoid is the most common phytochemical type. Thus, the taxonomic significance of eremophilane sesquiterpenoids for the genus *Ligularia* needs further study in future. Most of these eremophilane sesquiterpenoids were obtained in the form of lactones, and they can be divided into five groups from the structural viewpoint: a) eremophilane-12,8-olides (**1-1** to **1-77**); b) eremophilane-12,8(14,6$\alpha$)-diolides (**1-78** to **1-99**); c) eremophilane-14,6$\alpha$-olides (**1-100** to **1-104**); d) furan-eremophilane sesquiterpenoids (**1-105** to **1-129**); e) other eremophilane sesquiterpenoids (**1-130** to **1-210**).

**1.1.1.1 Eremophilane-12,8-olides:** Eremophilane-12,8-olides (**1-1** to **1-77**) are the most popular eremophilane lactones. Compounds **1-1** to **1-36** are eremophilane-12,8$\alpha$-olides, while compounds **1-37** to **1-57** are eremophilane-12,8$\beta$-olides. Of the structures **1-1** to **1-77**, $H_{a/b}$-6, H-8, and H-10 were always substituted by various substitutions, such as OH, OAc, OAng, OMe, and OEt. In some cases (**1-5**, **1-8**, **1-12**, **1-13**, **1-16 to 1-18**, **1-22**, **1-24 to 1-26**, and **1-31** to **1-35**), an epoxy group has been formed between C-1 and C-10. Furthermore, a double bond is often constructed between C-8 and C-9 (**1-58** to **1-73**) or C-7 and C-8 (**1-74** to **1-77**).

**1.1.1.2 Eremophilane-12,8(14,6*α*)-diolides:** Of such structures (**1-78** to **1-99**), an interesting phenomenon is that all H-6 protons are *β*-oriented. In addition, the H-8 protons are often substituted by OH, OMe, or OEt, while a double bond is often constructed between C-8 and C-9 in some cases (**1-91** to **1-94** and **1-99**).

**1.1.1.3 Eremophilane-14,6*α*-olides:** As that of **1-78** to **1-99**, the H-6 protons in structures **1-100** to **1-104** are all *β*-oriented. This phenomenon may show some relationships with the biosynthetic pathway of 14,6*α*-olide moiety.

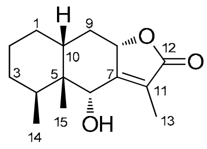

**1-1**

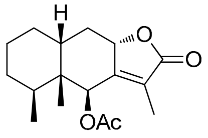

**1-2**

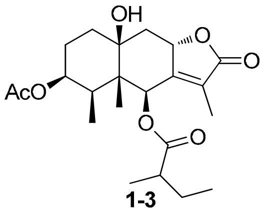

**1-3**

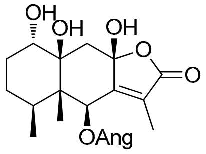

**1-4**

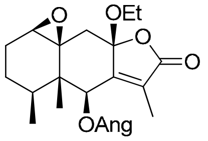

**1-5**

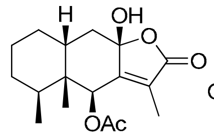

**1-6**

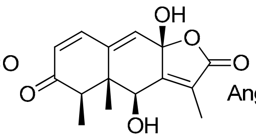

**1-7**

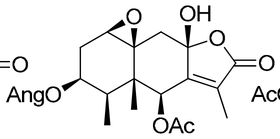

**1-8**

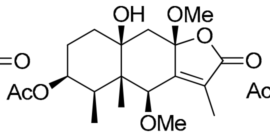

**1-9**

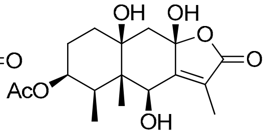

**1-10**

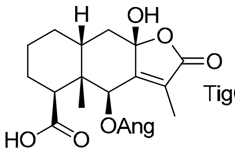

**1-11**

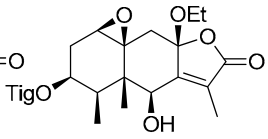

**1-12**

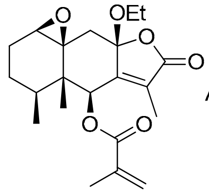

**1-13**

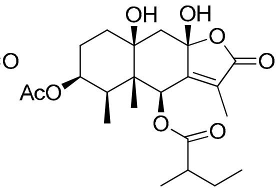

**1-14**

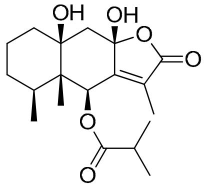

**1-15**

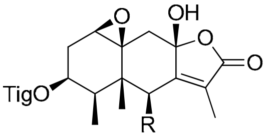

| | R |
|---|---|
| **1-16** | OAc |
| **1-17** | OH |
| **1-18** | H |

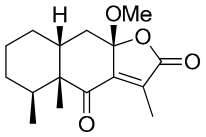

**1-19**

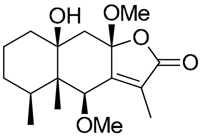

**1-20**

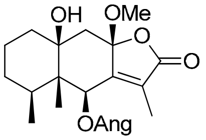

**1-21**

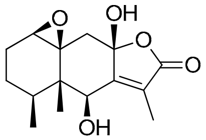

**1-22**

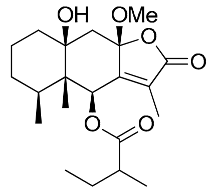

**1-23**

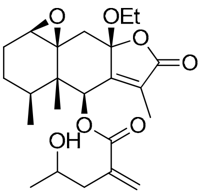

**1-24**

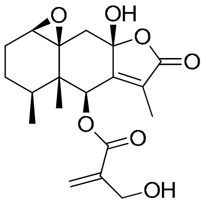

**1-25**

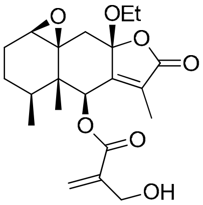

**1-26**

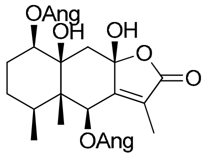

**1-27**

**1.1.1.4 Furan-eremophilane Sesquiterpenoids:** All of structures **1-105** to **1-129** possess a furan ring. Due to the sructural similarity, these compounds are put in one group in this review. Their most obvious structural characteristic is that the C-6 positions always possess various substitutions, such as OH, OAc, and OAng.

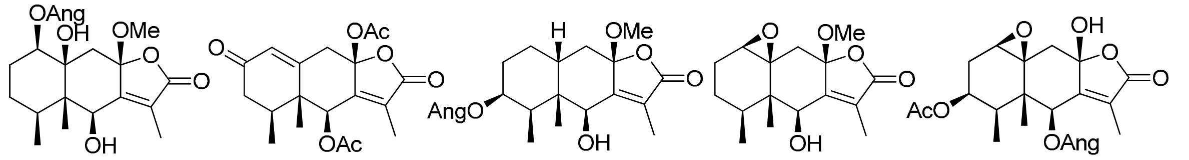

1-28 1-29 1-30 1-31 1-32

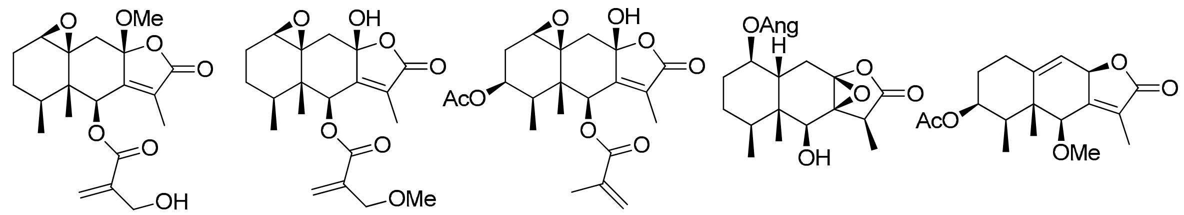

1-33 1-34 1-35 1-36 1-37

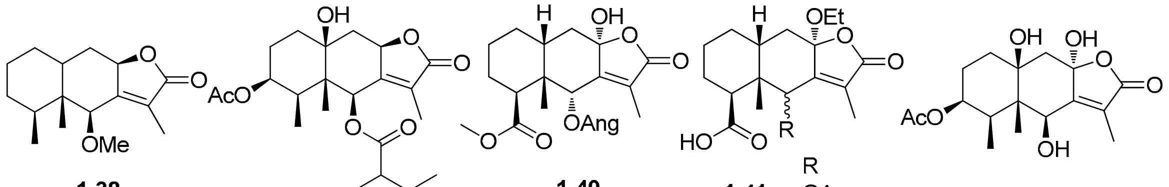

1-38 1-39 1-40

R
1-41 $\alpha$-OAng
1-42 $\beta$-OAng

1-43

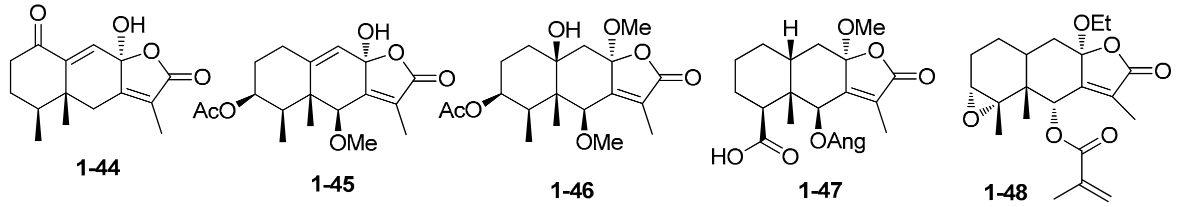

1-44 1-45 1-46 1-47 1-48

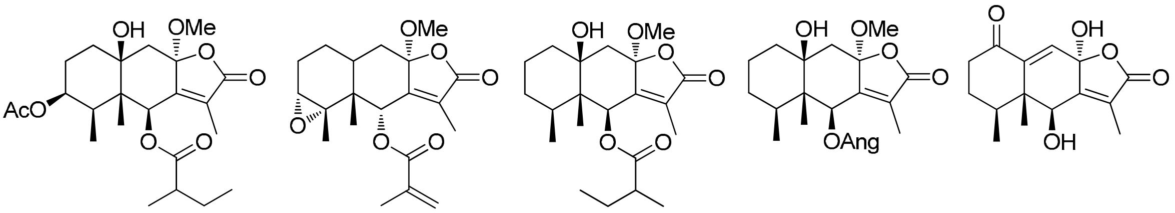

1-49 1-50 1-51 1-52 1-53

**1.1.1.5 Other Eremophilane Sesquiterpenoids:** Besides above structures, there are still 81 eremophilane-type sesquiterpenoids (**1-130**–**1-210**) covered here. Compounds **1-133** to **1-136** are isolated from the roots of *L. fischeri* in our lab[14,54], and **1-135** and **1-136** are obtained as a pair of epimers and their atructures have been confirmed by single-crystal X-ray diffraction analysis[14]. Compound **1-137** is obtained as a sesquiterpenoid lactam[3], which is rarely discovered from nature source. The structures **1-200** to **1-210** represent a rare carbon skeleton, and the probable biosynthetic pathway of such skeleton is proposed[77,78].

**1.1.2 Bisabolane Sesquiterpenoids:** Bisabolane sesquiterpenoids **1-211** to **1-242** and their corresponding plant sources were indicated in Table 1. Among them, $H_{a/b}$-1, H-2, $H_{a/b}$-8, and $H_{a/b}$-10 are always substituted by OH or OAng. Furthermore, there is often an epoxy group formed between C-3 and C-4 (**1-213**, **1-214**, **1-218**, **1-219**, **1-227** to **1-231**, and **1-**

**235**) or between C-10 and C-11 (**1-214**, **1-215**, **1-217** to **1-220**, **1-223**, **1-226** to **1-229**, **1-233**, and **1-235**). In some cases (**1-237** to **1-242**), ring A is often oxygenated to benzonic moiety.

**1.1.3 Oplopane Sesquiterpenoids:** The six oplopane-type sesquiterpenoids **1-243** to **1-248**, listed in Table 1, were all isolated from the roots of *L. narynensis*. Considering the structural characteristics, the C-3, C-4, C-8, and C-9 positions often possess various substitutions. Furthermore, in all of these

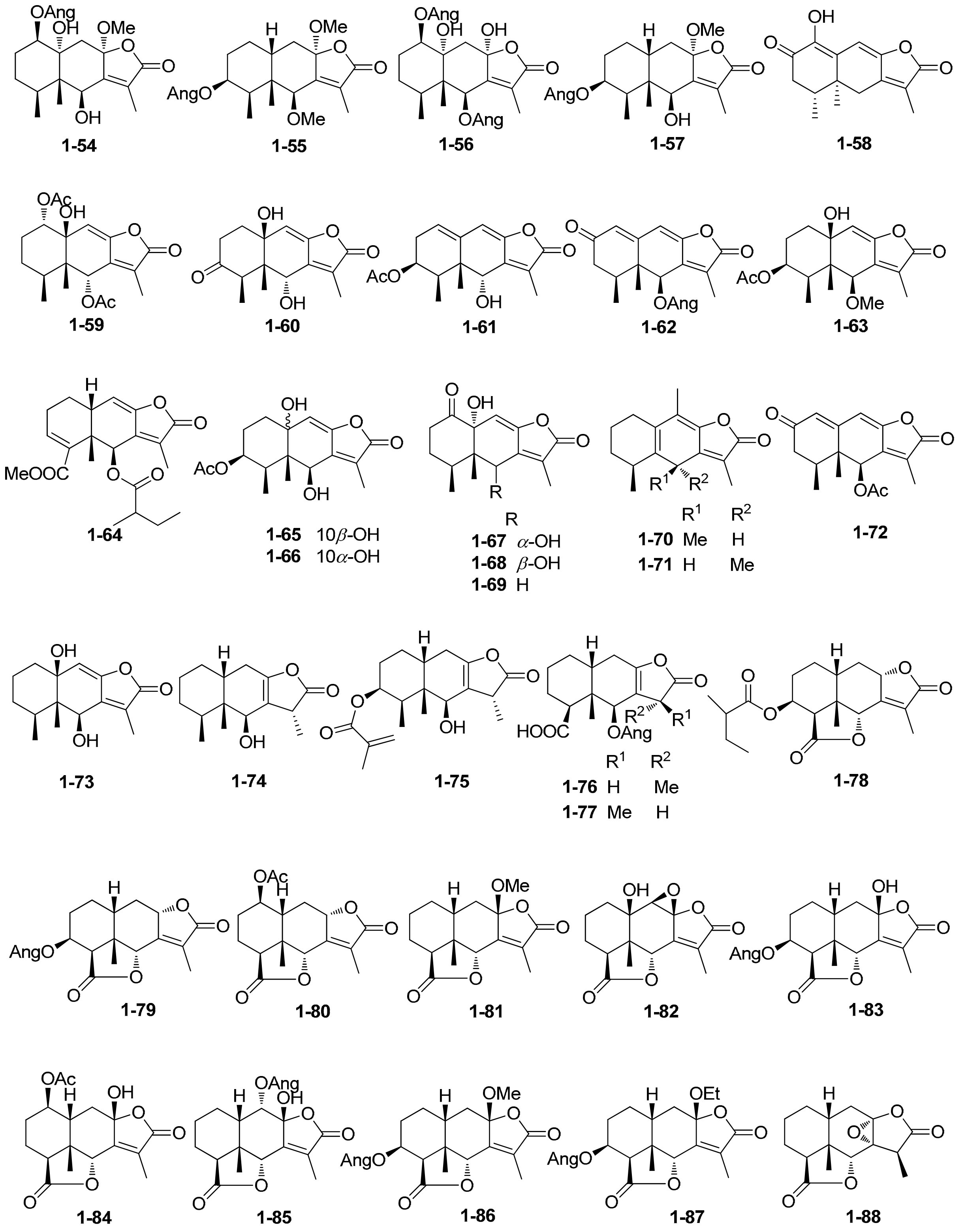

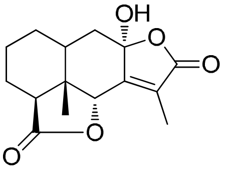

**1-89**

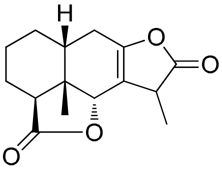

**1-90**

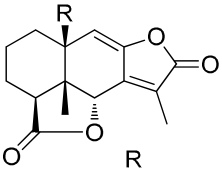

| | R |
|---|---|
| **1-91** | H |
| **1-92** | OH |

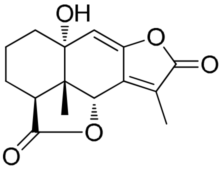

**1-93**

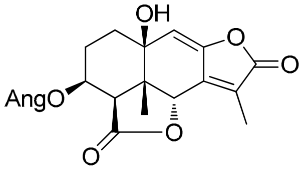

**1-94**

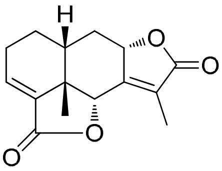

**1-95**

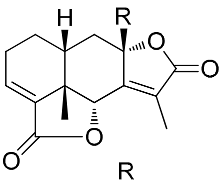

| | R |
|---|---|
| **1-96** | OH |
| **1-97** | OMe |
| **1-98** | OEt |

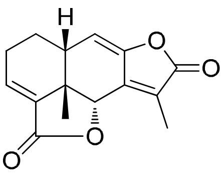

**1-99**

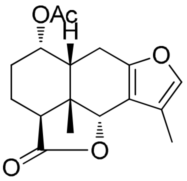

**1-100**

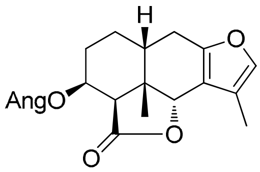

**1-101**

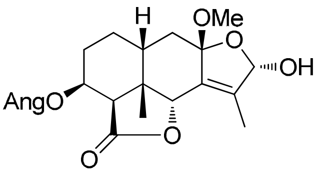

**1-102**

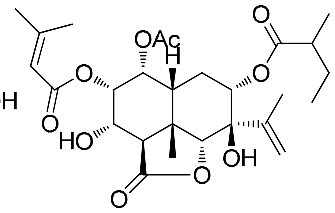

**1-103**

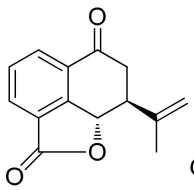

**1-104**

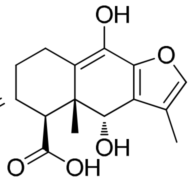

**1-105**

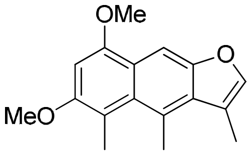

**1-106**

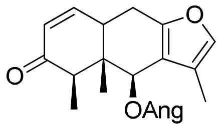

**1-107**

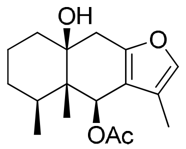

**1-108**

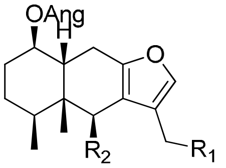

| | R1 | R2 |
|---|---|---|
| **1-109** | OH | H |
| **1-110** | H | OH |
| **1-111** | H | H |

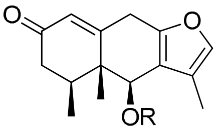

| | R |
|---|---|
| **1-112** | Ang |
| **1-113** | Ac |
| **1-114** | H |

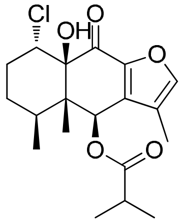

**1-116**

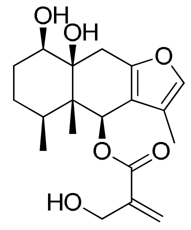

**1-115**

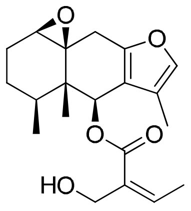

**1-117**

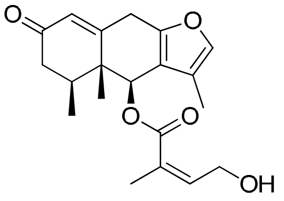

**1-118**

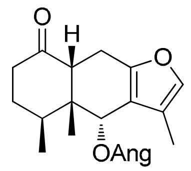

**1-119**

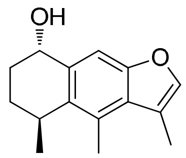

**1-120**

structures, there is an epoxy group posited between C-11 and C-12.

**1.1.4 Other Sesquiterpenoids:** Besides the above main sesquiterpenoids types, there were still guaiane-types **1-249** to **1-251**, pseudoguaiane-types **1-252** and **1-253**, and eudesmane-types **1-254** to **1-258**, as well as other types **1-259** to **1-267** being reviewed. Their names and corresponding plant sources were detailed in Table 1.

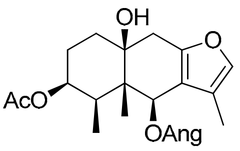

**1-121**

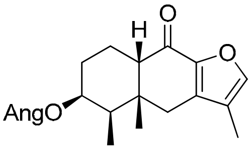

**1-122**

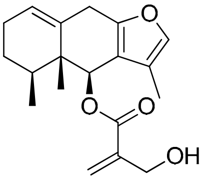

**1-123**

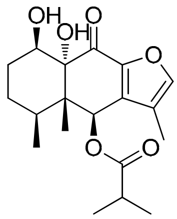

**1-124**

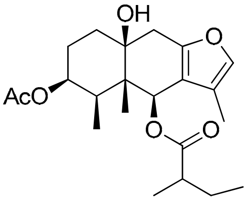

**1-125**

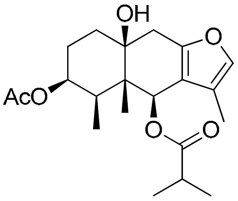

**1-126**

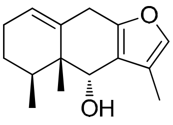

**1-127**

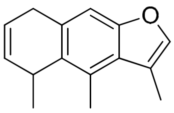

**1-128**

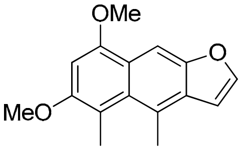

**1-129**

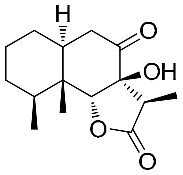

**1-130**

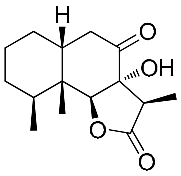

**1-131**

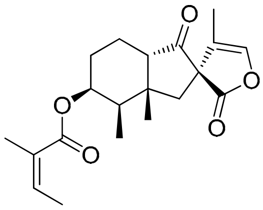

**1-132**

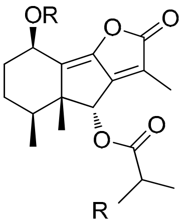

**1-133** H
**1-134** Ac

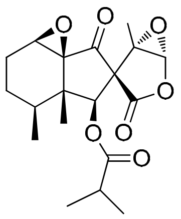

**1-135**

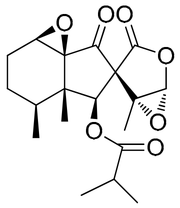

**1-136**

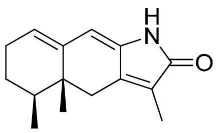

**1-137**

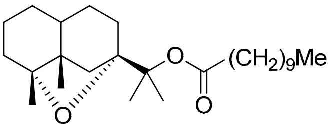

**1-138**

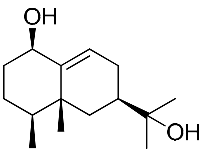

**1-139**

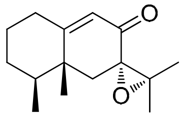

**1-140**

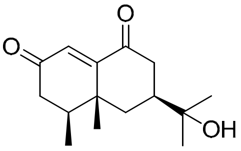

**1-141**

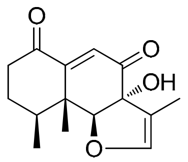

**1-142**

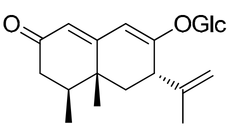

**1-143**

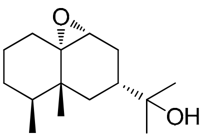

**1-144**

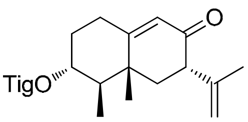

**1-145**

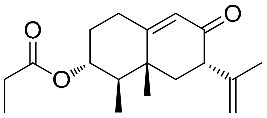

**1-146**

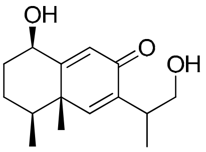

**1-147**

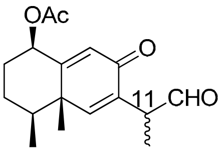

**1-148** 11*S
**1-149** 11*R

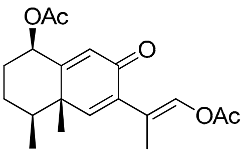

**1-150**

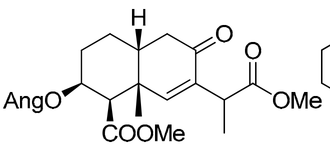

**1-151**

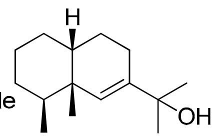

**1-152**

Compound **1-262**, possessing a new carbon skeleton, was discovered from *L. virgaurea* spp. *oligocephala*[9]. The lactones **1-263** and **1-264** were obtained as a pair of isomers, and their structres were determined using extensive spectroscopic methods[92]. The novel structures **1-265** and **1-266** were obtained as sesquiterpenoid-coumarin dimers[93], which are rarely discovered from nature source.

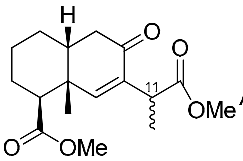

**1-153** 11*R
**1-154** 11*S

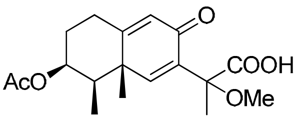

**1-155**

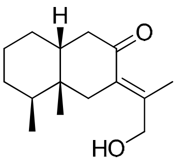

**1-156**

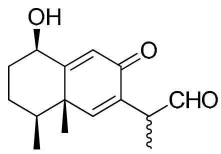

**1-157** 11 R*/S*

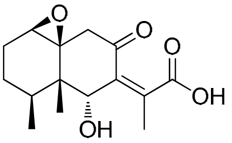

**1-158**

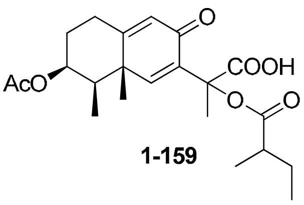

**1-159**

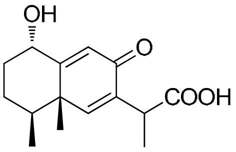

**1-160**

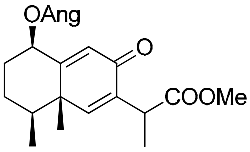

**1-161**

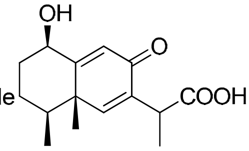

**1-164**

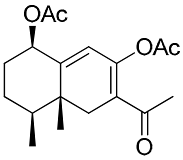

**1-165**

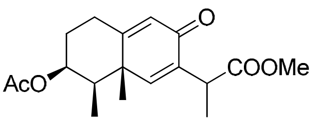

**1-162**

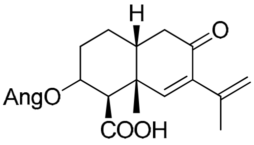

**1-163**

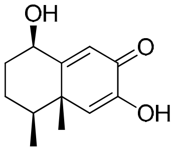

**1-166**

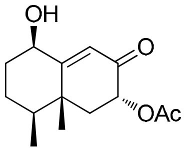

**1-167**

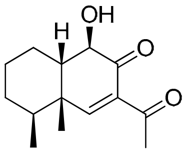

**1-169**

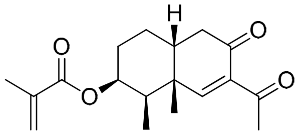

**1-168**

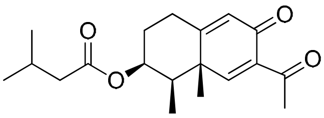

**1-170**

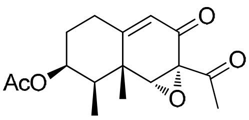

**1-171**

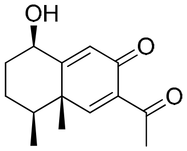

**1-172**

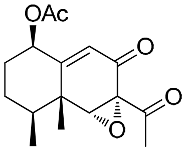

**1-173**

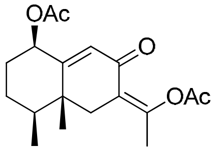

**1-174**

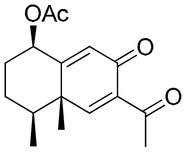

**1-175**

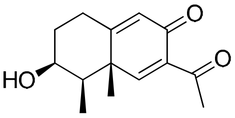

**1-176**

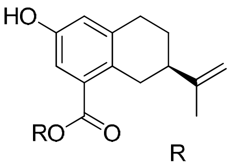

R
**1-177** Me
**1-178** Et

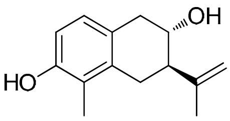

**1-179**

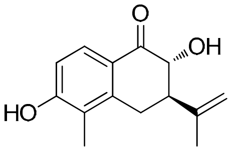

**1-180**

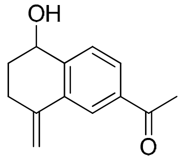

**1-181**

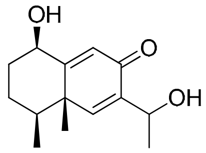

**1-182**

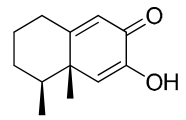

**1-183**

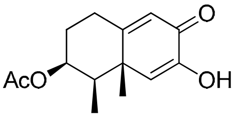

**1-184**

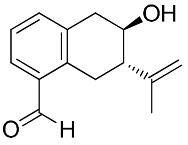

**1-185**

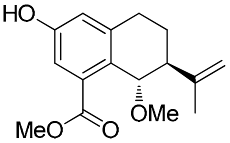

**1-186**

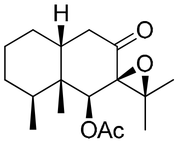

**1-187**

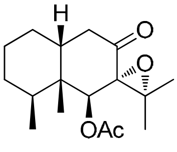

**1-188**

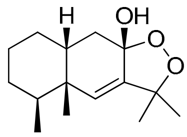

**1-189**

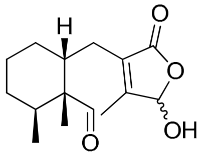

**1-190**

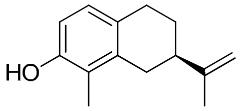

**1-191**

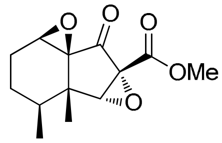

**1-192**

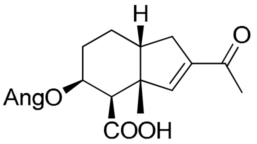

**1-193**

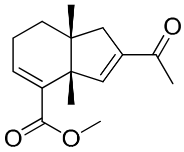

**1-194**

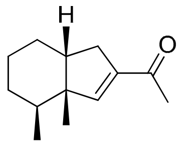

**1-195**

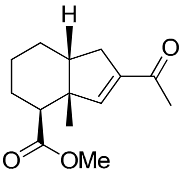

**1-196**

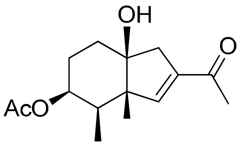

**1-197**

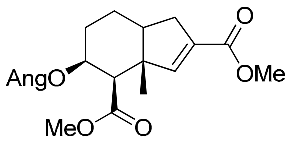

**1-198**

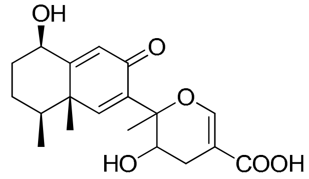

**1-199**

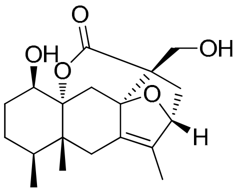

**1-200**

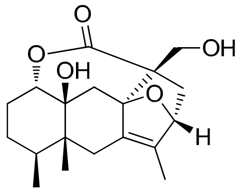

**1-201**

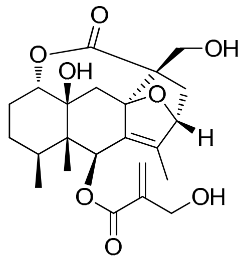

**1-202**

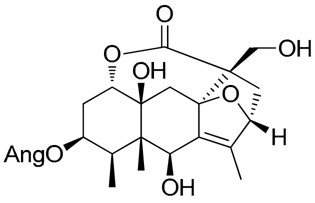

**1-203**

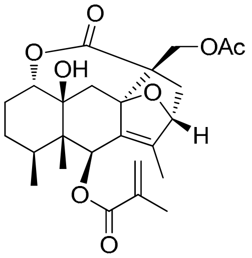

**1-204**

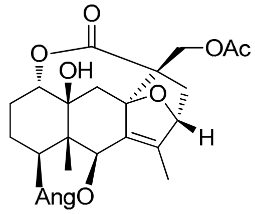

**1-205**

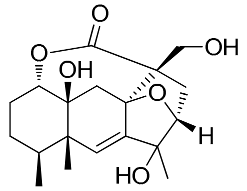

**1-206**

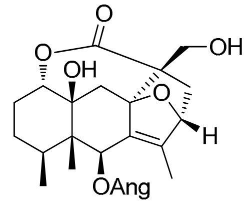

**1-207**

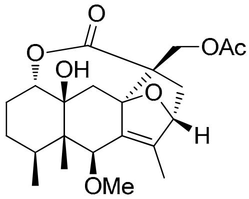

**1-208**

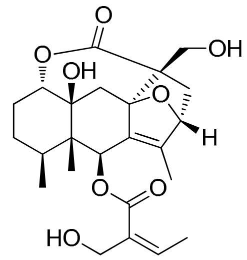

**1-209**

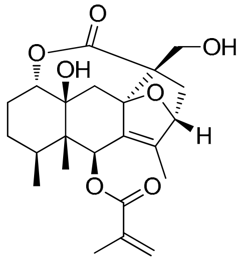

**1-210**

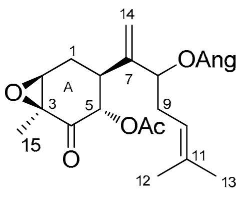

**1-211**

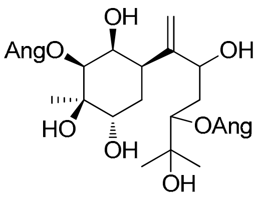

**1-212**

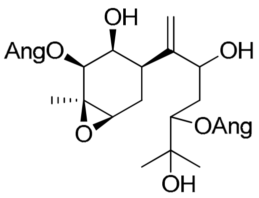

**1-213**

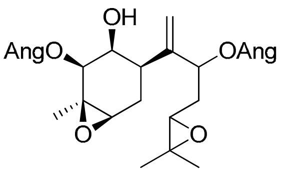

**1-214**

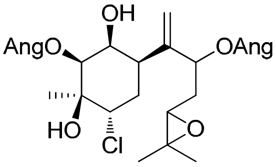

1-215

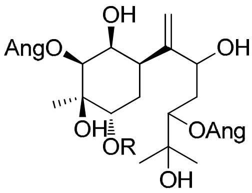

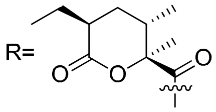

1-216

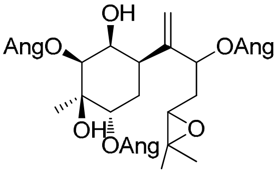

1-217

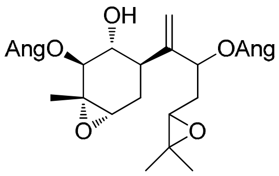

1-218

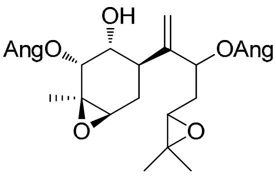

1-219

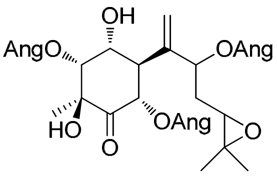

1-220

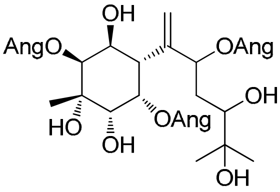

1-221

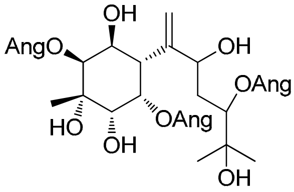

1-222

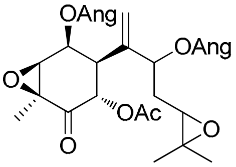

1-223

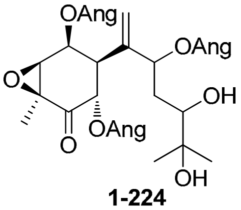

1-224

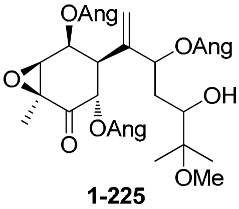

1-225

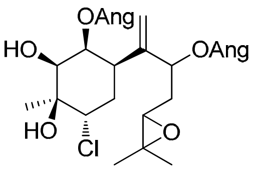

1-226

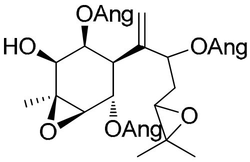

1-227

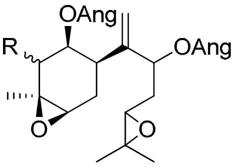

| | R |
|---|---|
| 1-228 | β-OAng |
| 1-229 | 2H |

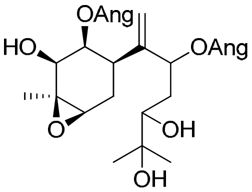

1-230

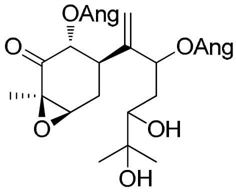

1-231

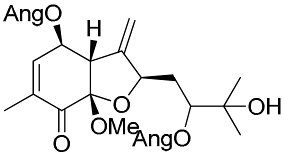

1-232

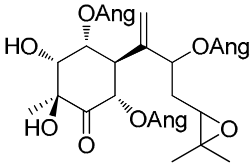

1-233

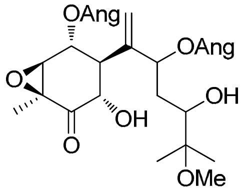

1-234

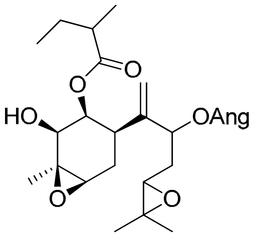

1-235

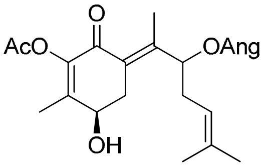

1-236

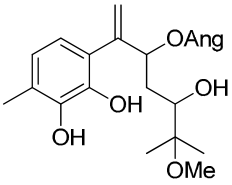

1-237

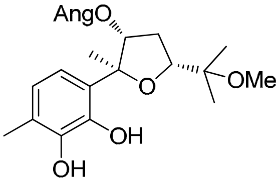

1-238

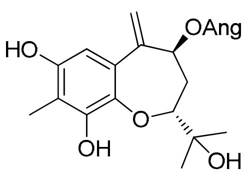

**1-239**

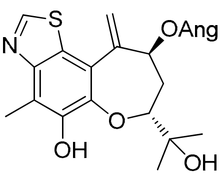

**1-240**

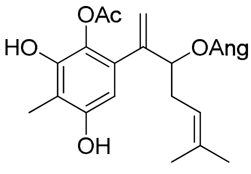

**1-241**

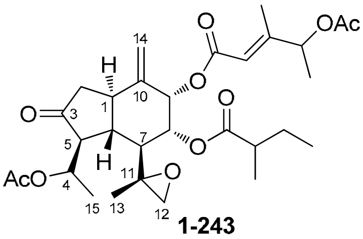

**1-243**

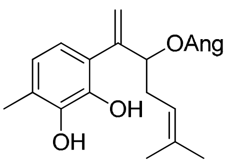

**1-242**

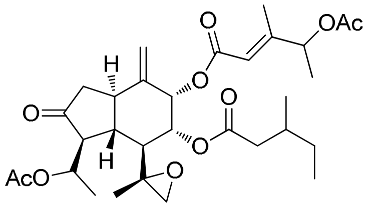

**1-244**

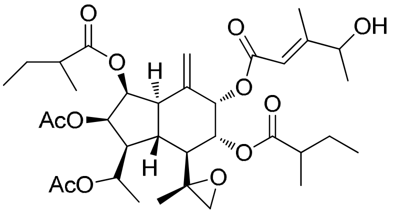

**1-245**

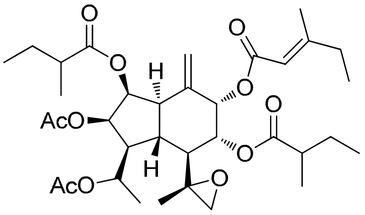

**1-246**

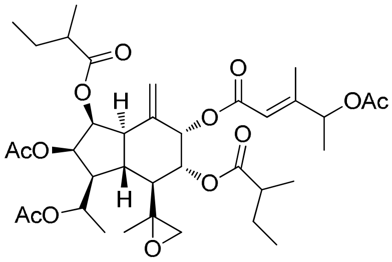

**1-247**

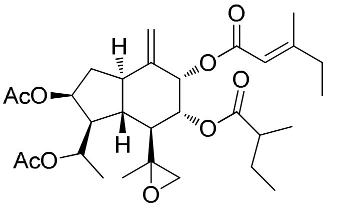

**1-248**

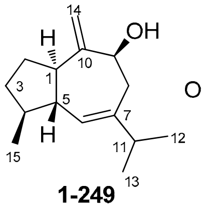

**1-249**

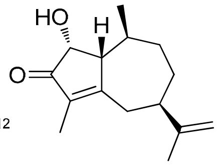

**1-250**

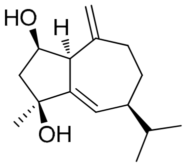

**1-251**

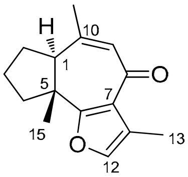

**1-252**

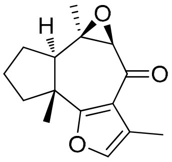

**1-253**

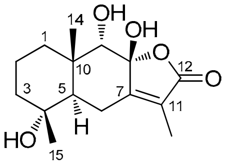

**1-254**

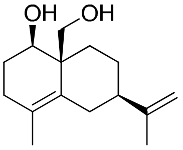

**1-255**

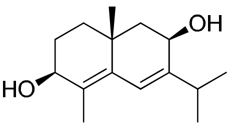

**1-256**

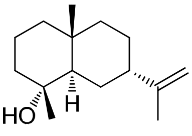

**1-257**

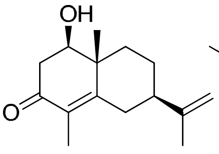

**1-258**

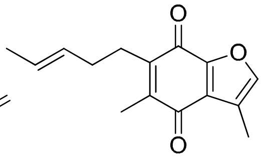

**1-259**

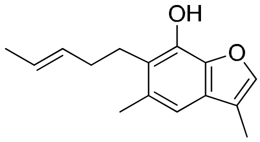

| | R |
|---|---|
| **1-260** | OH |
| **1-261** | OMe |

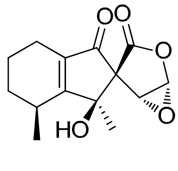

**1-262**

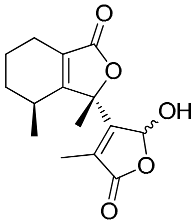

**1-263**

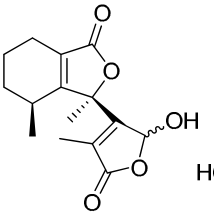

**1-264**

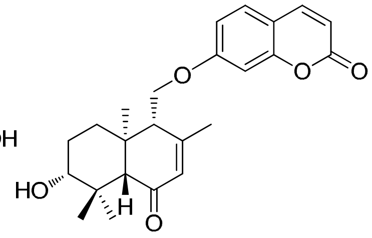

**1-265**

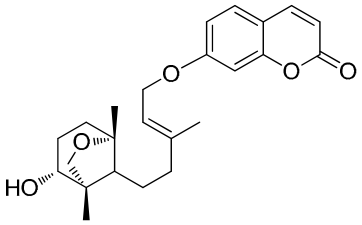

**1-266**

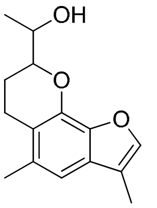

**1-267**

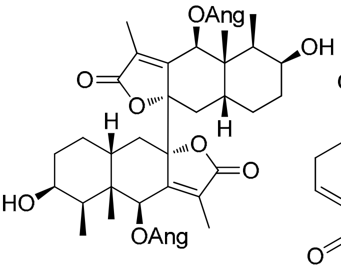

**1-268**

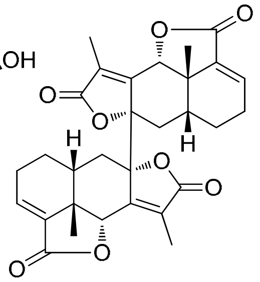

**1-269**

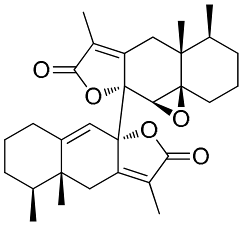

**1-270**

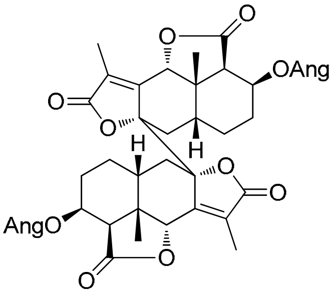

**1-271**

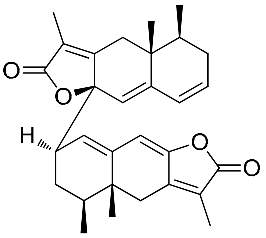

**1-272**

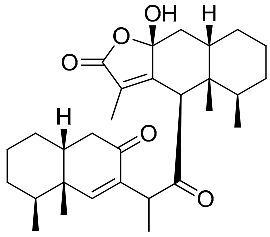

**1-273**

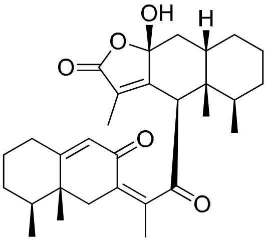

**1-274**

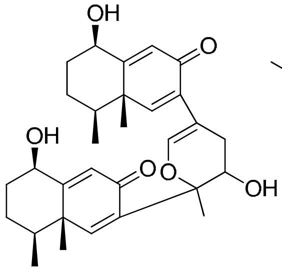

**1-275**

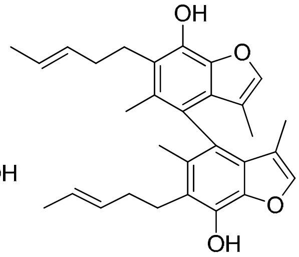

**1-276**

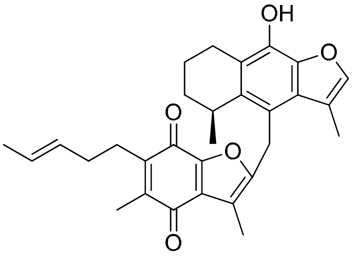

**1-278**

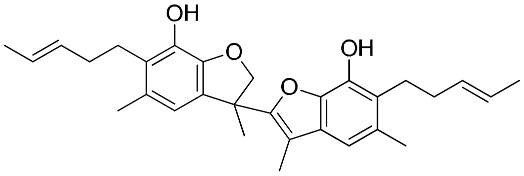

**1-277**

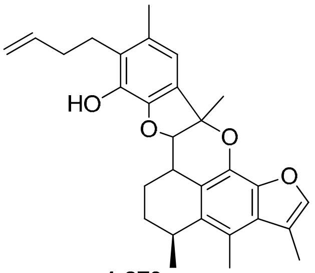

**1-279**

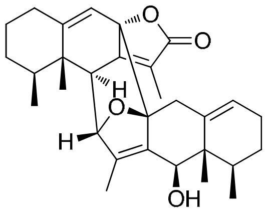

**1-280**

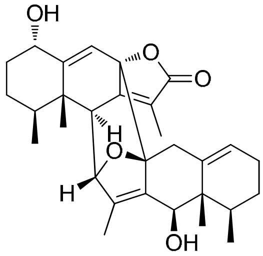

**1-281**

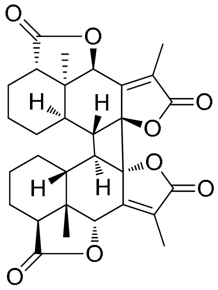

**1-282**

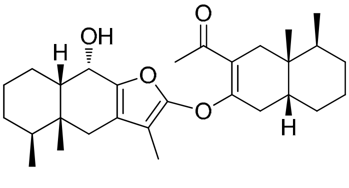

**1-283**

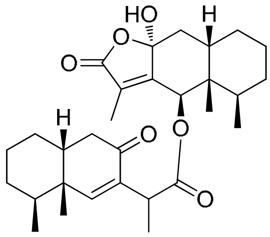

**1-285**

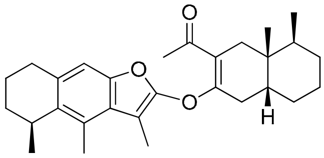

**1-284**

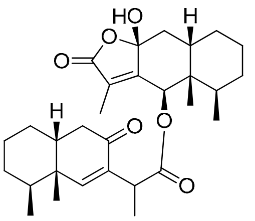

**1-286**

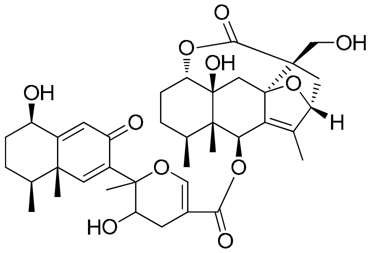

**1-287**

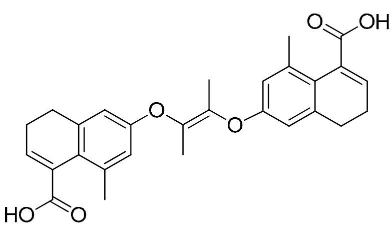

**1-288**

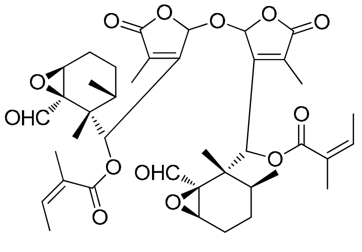

**1-289**

**1.1.5 Sesquiterpenoid Dimers:** The 22 sesquiterpenoid dimers **1-268** to **1-289** have been indicated in Table 1. Of these, structures **1-268** to **1-282** share the C-C linkage pattern, while **1-283** to **1-289** share the C-O-C linkage pattern[1,3,31,38,44,53,57,63,77,95–99].

**1.2 Monoterpenoids and Diterpenoids:** The structures of monoterpenoids **2-1** and **2-2** and diterpenoids **2-3** to **2-5** were provided, and their names and plant sources were listed in Table 2[15,62,88,100]. Of them, structure **2-3** was isolated as a $C_{19}$-diterpenoid carbon skeleton from *L. sagitta*, and its structure was further confirmed using single-crystal X-ray diffraction method[62].

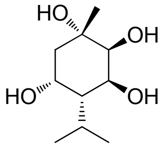

**2-1**

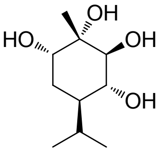

**2-2**

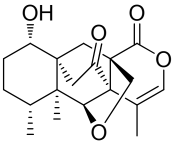

**2-3**

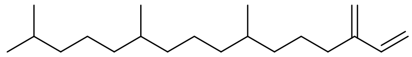

**2-4**

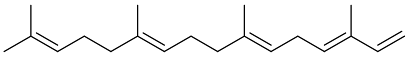

**2-5**

**1.3 Triterpenoids:** The ten triterpenoids **3-1** to **3-10** mainly comprise oleane and norursane types. Their names and corresponding plant sources were indicated in Table 3[40,47,100–105]. Of them, compounds **3-3** and **3-4** were obtained as triterpenoid saponins from *L. veitchiana*[102,103]. Compounds **3-5** and **3-6** were isolated from *L. intermedia* in the form of 3,4-seco-oleanolic triterpene acids[104]. Compounds **3-7** and **3-8** are norursane-type triterpenoids and were isolated from *L. tongolensis*[47].

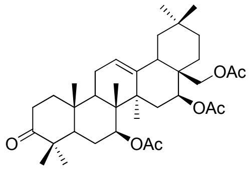

**3-1**

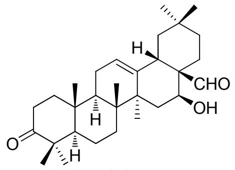

**3-2**

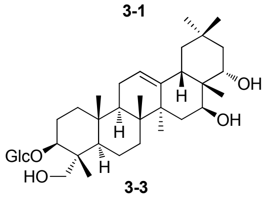

**3-3**

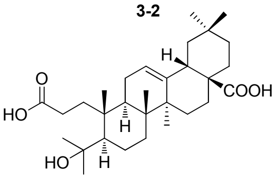

**3-5**

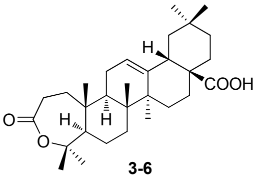

**3-6**

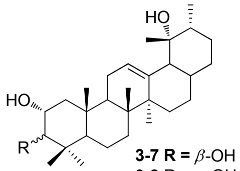

**3-7** R = β-OH
**3-8** R = α-OH

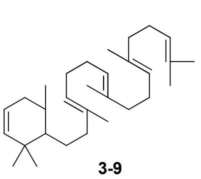

**3-9**

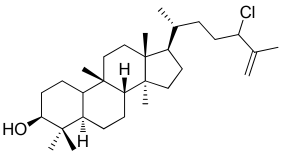

**3-10**

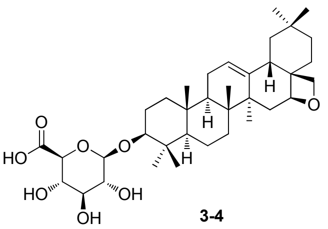

**3-4**

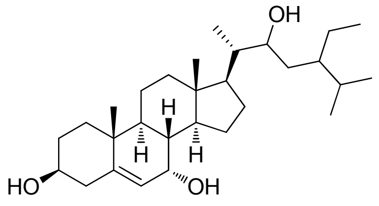

**4-1**

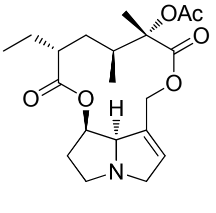

**4-2**

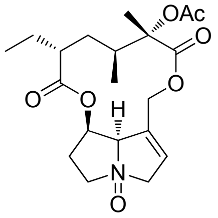

**4-3**

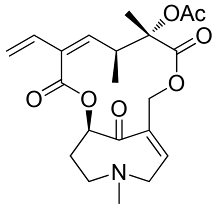

**4-4**

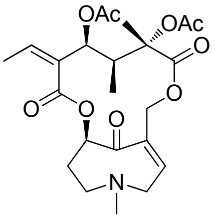

**4-5**

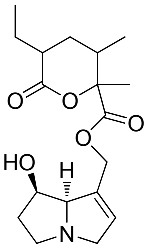

**4-6**

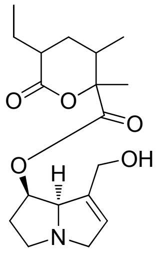

**4-7**

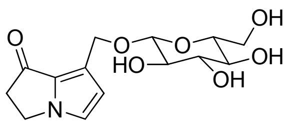

**4-8**

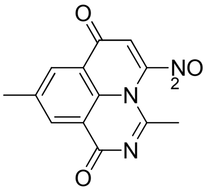

**4-9**

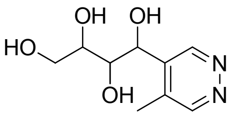

**4-10**

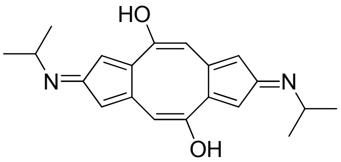

**4-11**

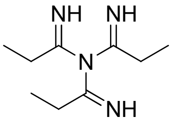

**4-12**

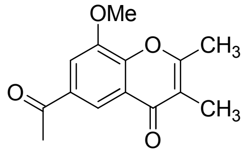

**4-13**

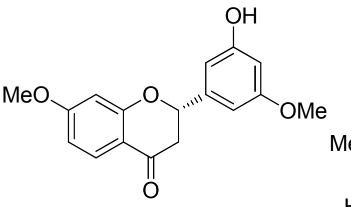

**4-14**

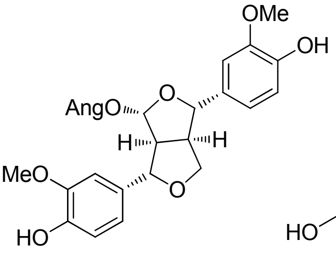

**4-15**

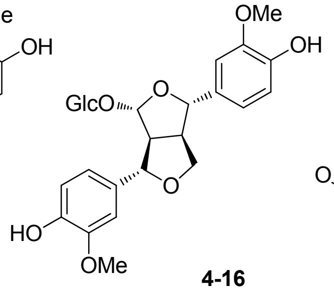

**4-16**

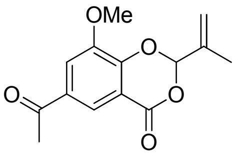

**4-18**

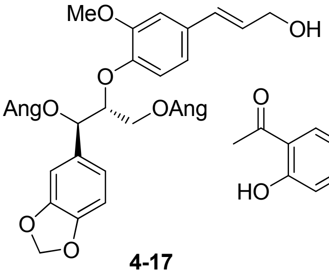

**4-17**

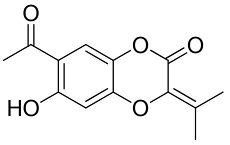

**4-19**

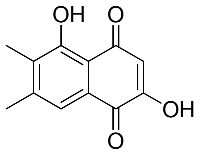

**4-20**

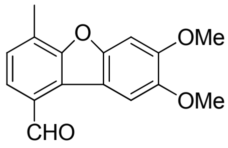

**4-21**

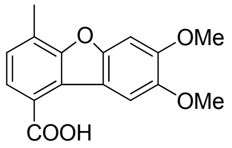

**4-22**

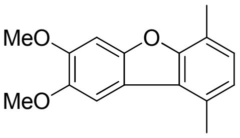

**4-23**

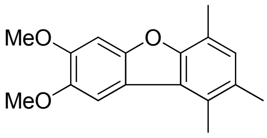

**4-24**

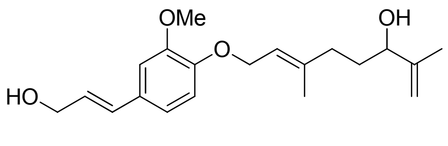

**4-25**

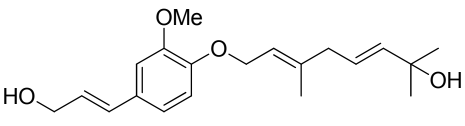

**4-26**

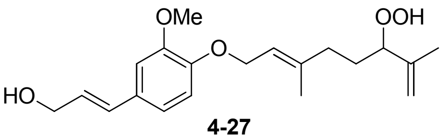

**4-27**

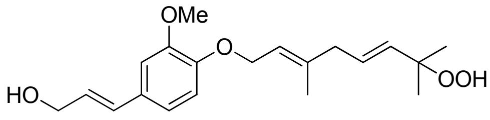

**4-28**

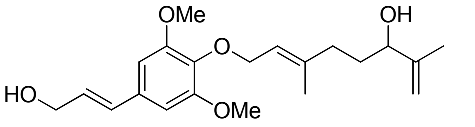

**4-29**

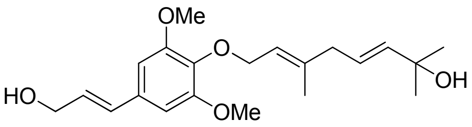

**4-30**

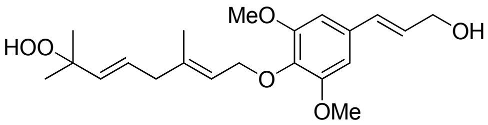

**4-31**

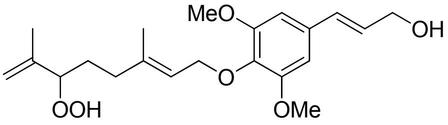

**4-32**

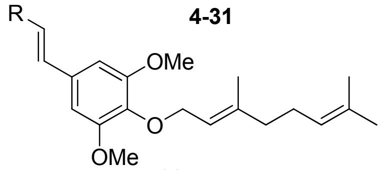

**4-33** R = $CH_2OH$
**4-34** R = CHO

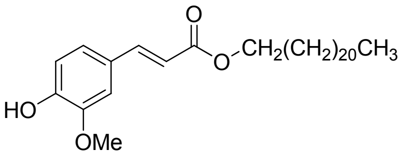

**4-35**

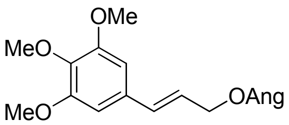

**4-36**

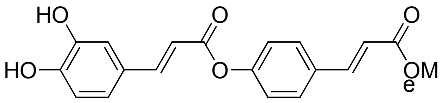

**4-37**

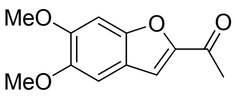

**4-38**

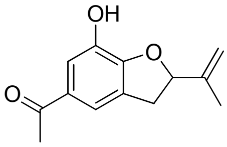

**4-39**

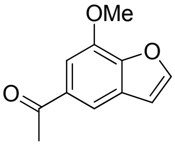

**4-40**

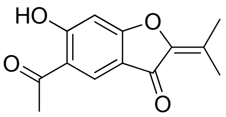

**4-41**

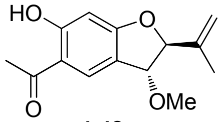

**4-42**

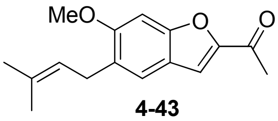

**4-43**

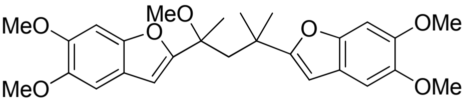

**4-44**

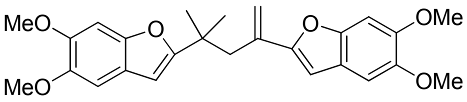

**4-45**

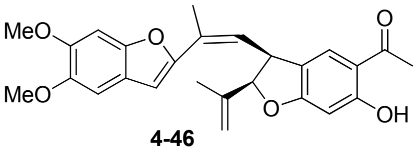

**4-46**

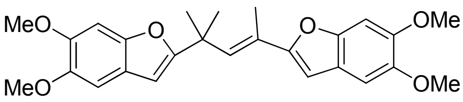

**4-47**

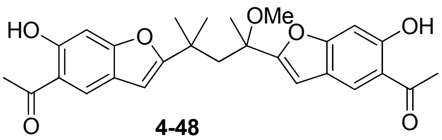

**4-48**

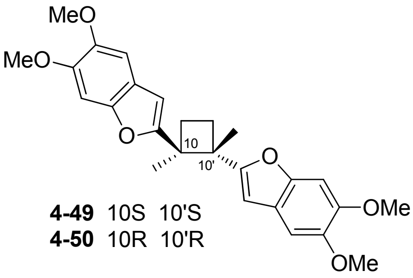

**4-49** 10S 10'S
**4-50** 10R 10'R

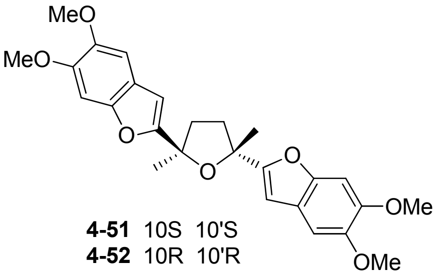

**4-51** 10S 10'S
**4-52** 10R 10'R

**1.4 Others:** Besides the above terpenoid constituents, there were still one steroid (**4-1**), 11 alkaloids (**4-2** to **4-12**), two flavonoids (**4-13** and **4-14**), and three lignans (**4-15** to **4-17**), as well as other secondary metabolites, possessing various skeletons (**4-18** to **4-64**), highlighted in Table 4[4,6,39,66,80,83,89,105–120]. Among these structures, **4-49** and **4-50**, **4-51** and **4-52**, **4-53** and **4-54** were isolated as racemates from *L. stenocephala*, which were further comfirmed by the chiral HPLC analysis[120].

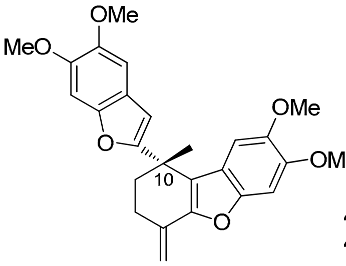

**4-53** 10S
**4-54** 10R

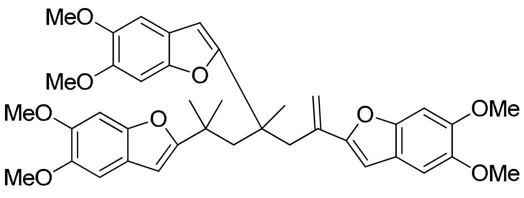

**4-55**

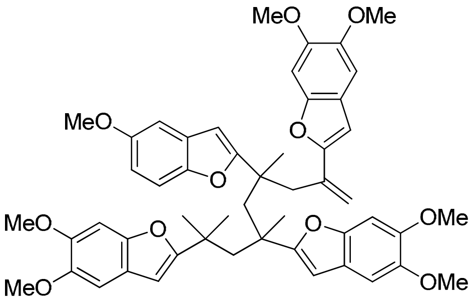

**4-56**

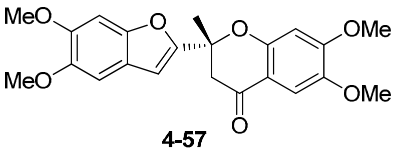

**4-57**

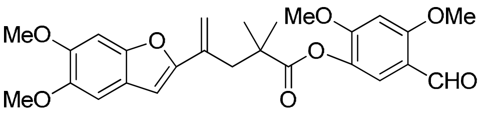

**4-58**

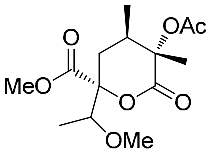

**4-59**

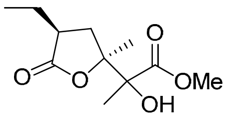

**4-60**

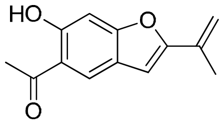

**4-61**

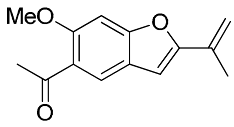

**4-62**

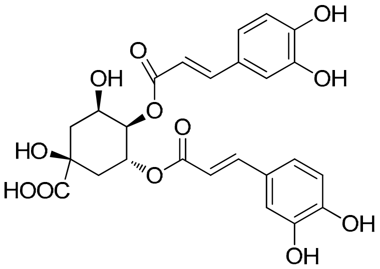

**4-63**

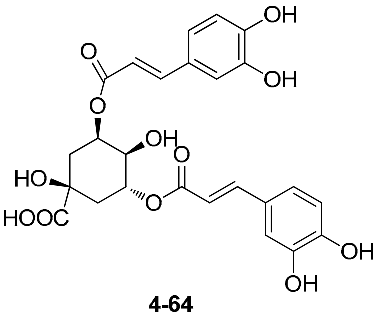

**4-64**

**Table 1. Sesquiterpenoids from the genus *Ligularia***

| No. | compound name | plant source | part of plant | Ref. |
|---|---|---|---|---|
| **1-1** | (6*α*,8*α*)-6-Hydroxyeremophil-7(11)-en-12,8-olide | *L. muliensis* | root | [15] |
| **1-2** | (6*α*,8*α*)-6-Acetyloxyeremophil-7(11)-en-12,8-olide | *L. muliensis* | root | [15] |
| **1-3** | 3*β*-Aacetyloxy-6*β*-(2-methylbutanoyloxy)-10*β*-hydroxyeremophil-7(11)-en-12,8*α*-olide | *L. japonica* | root | [16] |
| **1-4** | 6*β*-Angeloyloxy-1*α*,8*β*,10*β*-Trihydroxyeremophil-7(11)-en-12,8*α*-olide | *L. virgaurea* | root | [17] |
| **1-5** | 6*β*-Angeloyloxy-1*β*,10*β*-epoxy-8*β*-ethoxyeremophil-7(11)-en-12,8*α*-olide | *L. virgaurea* | root | [17] |

| | | | | |
|---|---|---|---|---|
| **1-6** | (6β,8α)-6-Acetyloxy-8-hydroxyeremophil-7(11)-en-12,8-olide | *L. muliensis* | root | [15] |
| **1-7** | No Name | *L. veitchiana* | root | [18] |
| **1-8** | 1β,10β-Epoxy-6β-acetoxy-3β-angeloyloxy-8β-hydroxy-eremophil-7(11)-en-8,12α-olide | *L. sagitta* | aerial part | [19] |
| **1-9** | 3β–Acetoxy–10β–hydroxy–6β,8β–dimethoxy ‑ eremophil–7(11)–en–12,8α–olide | *L. duciformis* | whole plant | [20] |
| **1-10** | 3β-Acetoxy-6β,8β,10β-trihydroxyeremophil-7(11)-en-12,8α-olide | *L. duciformis* | whole plant | [20] |
| **1-11** | 8β-Hydroxy-6β-angeloyloxyeremophil-7(11)-en-8α,12-olide-15-oic acid | *L. przewalskii* | root | [21] |
| **1-12** | (1β,3β,6β,8β,10β)-3-Angeloyloxy-1,10-epoxy-8-ethoxy-6-hydroxyeremophil-7(11)-en-8,12α-olide | *L. sagitta* | aerial part | [22] |
| **1-13** | 1β,10β-Epoxy-8β-ethoxy-6β-((2'-methylacryloyl)oxy)eremophil-7(11)-en-12,8α-olide | *L. virgaurea* | root | [17] |
| **1-14** | (3β,6β,8β,10β)-3-Acetyl-8,10-dihydroxy-6-(2-methyl-1-oxobutoxy)eremophil-7(11)-eno-12,8-lactone | *L. fischeri* | root | [23] |
| **1-15** | 8β,10β-Dihydroxy-6β-isobutyryloxyeremophil-7(11)-en-12,8-olide | *L. kanaitzensis* | root | [24] |
| **1-16** | (1β,3β,6β,8β,10β)-6-Acetoxy-3-angeloyloxy-1,10-epoxy-8-hydroxyeremophil-7(11)-en-8,12α-olide | *L. sagitta* | aerial part | [22] |
| **1-17** | (1β,3β,6β,8β,10β)-3-Angeloyloxy-1,10-epoxy-6,8-dihydroxyeremophil-7(11)-en-8, 12α-olide | *L. sagitta* | aerial part | [22] |
| **1-18** | (1β,3β,8β,10β)-3-Angeloyloxy)-1,10-epoxy-8-hydroxyeremophil-7(11)-en-8,12α-olide | *L. sagitta* | aerial part | [22] |
| **1-19** | (–)-6-Oxoeremophilenolide | *L. fischeri* var. *spiciformis* | leaves | [25] |
| **1-20** | 6β,8β-Dimethoxy-10β-hydroxyeremophil-7(11)-en-12,8α-olide | *L. sagitta* | root | [26] |
| **1-21** | 6β-Angeloyloxy-10β-hydroxy-8β-methoxy-eremophil-7(11)-en-12,8α-olide | *L. sagitta* | root | [26] |
| **1-22** | 1β,10β-Epoxy-6β,8β-dihydroxy-eremophil-7(11)-en-12, 8α-olide | *L. veitchiana* | whole plant | [27] |
| **1-23** | 6β-(2'-Methylbutanoyloxy)-10β-hydroxy-8β-methoxyeremophil-7(11)-en-12,8α-olide | *L. sagitta* | root | [26] |
| **1-24** | 1β,10β-Epoxy-6β-(2'-semialdehydeacetal-hydroxymethylacryloyloxy)-8β-ethoxy-eremophil-7(11)-en-12,8α-olide | *L. veitchiana* | whole plant | [28] |
| **1-25** | 1β, 10β-Epoxy-6β(2'-hydroxymethylacryloyloxy)-8β-hydroxy-eremophila-7(11)-en-12,8α-olide | *L. veitchiana* | root | [29] |
| **1-26** | 1β, 10β-Epoxy-6β(2'-hydroxymethylacryloyloxy)-8β-ethoxy-eremophila-7(11)-en-12,8α-olide | *L. veitchiana* | root | [29] |
| **1-27** | 1β,6β-Diangeloyloxy-8β,10β-dihydroxyeremophil-(11)-en-8α,12-olide | *L. myriocephala* | whole plant | [30] |
| **1-28** | 1β-Angeloyloxy-6β,10β-dihydroxy-8β- methoxyeremophila-7(11)-en-8α,12-olide | *L. myriocephala* | whole plant | [13] |
| **1-29** | 6β,8β-Diacetyl-2-oxoeremophila-1(10),7(11)-dien-12,8-olide | *L. virgaurea* spp. *oligocephala* | whole plant | [31] |
| **1-30** | 3β-Angeloyloxy-6β-hydroxy-8β-methoxyeremophil-7(11)-en-12,8α-olide | *L. hiberniflorum* | rhizome | [32] |
| **1-31** | 1β,10β-Epoxy-6β-hydroxy-8β-methoxy-eremophil-7(11)-en-12,8α-olide | *L. veitchiana* | root | [33] |
| **1-32** | 1β,10β-Epoxy-3β-acetoxy-6β-angeloyloxy-8β-hydroxy-eremophil-7(11)-en-12,8α-olide | *L. veitchiana* | root | [33] |
| **1-33** | 1β,10β-Epoxy-6β-(2'-hydroxymethylacrylyloxy)-8β-methoxy-eremophil-7(11)-en-12,8α-olide | *L. veitchiana* | root | [33] |
| **1-34** | 1β,10β-Epoxy-6β-(2'-methoxymethylacrylyloxy)-8β-hydroxy-eremophil-7(11)-en-12,8α-olide | *L. veitchiana* | root | [33] |
| **1-35** | 1β,10β-Epoxy-3β-acetoxy-6β-(2'-methylacrylyloxy)-8β-hydroxy-eremophil-7(11)-en-12,8α-olide | *L. veitchiana* | root | [33] |
| **1-36** | Subspicatin D | *L. subspicata* | root | [34] |
| **1-37** | 3β-Acetoxy-6β-methoxyeremophila-7(11),9(10)-dien-12,8β-olide | *L. duciformis* | whole plant | [20] |
| **1-38** | 6β-Methoxyeremophil-7(11)-en-8β,12-olide | *L. virgaurea* | root | [35] |
| **1-39** | 6β-(2'-Methylbutanoyloxy)-3β-acetoxy-10β-hydroxy-8α-methoxyeremophil-7(11)-en-8β,12-olide | *L. hodgsonii* | root and aerial part | [36] |
| **1-40** | (4*S*,5*S*,6*R*,8*R*,10*R*)-6-Angeloyloxy-8-hydroxyeremophil-7(11)-en-8,12-olide-15-carboxylic acid methyl ester | *L. hodgsonii* | root and rhizome | [37] |
| **1-41** | (4*S*,5*S*,6*R*,8*R*,10*R*)-6-Angeloyloxy-8-ethoxyeremophil-7(11)-en-8,12-olid-15-oic acid | *L. hodgsonii* | root and rhizome | [37] |
| **1-42** | (4*S*,5*S*,6*S*,8*R*,10*R*)-6-Angeloyloxy-8-ethoxyeremophil-7(11)-en-8,12-olid-15-oic acid | *L. hodgsonii* | root and rhizome | [37] |
| **1-43** | (3β,6β,8α,10β)-3-Acetyl-6,8,10-trihydroxyeremophil-7(11)-eno-12,8-lactone | *L. fischeri* | root | [23] |
| **1-44** | 8β-Hydroxy-1-oxo-(14α,15α-eremophil-7(11),9(10)-dien-12,8α-olide | *L. platyglossa* | root and rhizome | [38] |
| **1-45** | 3β-Acetoxy-8α-hydroxy-6β-methoxyeremophila-7(11),9(10)-dien-12,8β-olide | *L. duciformis* | whole plant | [20] |
| **1-46** | 3β-Acetoxy-10β-hydroxy-6β,8α-di-methoxyeremophil-7(11)-en-8β,12-olide | *L. hodgsonii* | root and aerial part | [36] |
| **1-47** | 8α-Methoxy-6β-angeloyloxyeremophil-7(11)-en-8β,12-olide-14-oic acid | *L. dolichobotrys* | whole plant | [39] |
| **1-48** | 3α,4α-Epoxy-6α-(2'-methylacryloyl)oxy-8α-ethoxyeremophil-7(11)-en-8β,12-olide | *L. virgaurea* | root | [35] |
| **1-49** | 6β-(2'-Methylbutanoyloxy)-3β-acetoxy-10β-hydroxyeremophil-7(11)-en-8β,12-olide | *L. hodgsonii* | root and aerial part | [36] |
| **1-50** | 3α,4α-Epoxy-6α-(2'-methylacryloyl)oxy-8α-methoxyeremophil-7(11)-en-8β,12-olide | *L. virgaurea* | root | [35] |
| **1-51** | 6β-(2'-Methylbutanoyloxy)-10β-hydroxy-8α-methoxyeremophil-7(11)-en-12,8β-olide | *L. sagitta* | root | [26] |
| **1-52** | 6β-Angeloyloxy-10β-hydroxy-8α-methoxyeremophil-7(11)-en-12,8β-olide | *L. sagitta* | root | [26] |
| **1-53** | 6β,8α-Dihydroxy-1-oxoeremophila-7(11),9(10)-diene-12,8-olide | *L. virgaurea* spp. *oligocephala* | whole plant | [9] |
| **1-54** | 1β-Angeloyloxy-6β,10α-dihydroxy-8α-methoxyeremophila-7(11)-en-8β,12-olide | *L. myriocephala* | whole plant | [13] |
| **1-55** | 3β-Angeloyloxy-6β,8α-dimethoxy-eremophil-7(11)-en-12,8β-olide | *L. hiberniflorum* | rhizome | [32] |
| **1-56** | 1β,6β-Diangeloyloxy-8α,10α-dihydroxy-eremophil-7(11)-en-8β,12-olide | *L. myriocephala* | whole plant | [30] |
| **1-57** | 3β-Angeloyloxy-6β-hydroxy-8α-methoxyeremophil-7(11)-en-12,8β-olide | *L. hiberniflorum* | rhizome | [32] |
| **1-58** | 1-Hydroxy-2-oxo-(14α,15α-eremophil-1(10),7(11),8(9)-trien-12,8-olide | *L. platyglossa* | root and rhizome | [38] |
| **1-59** | (4*R*,4a*S*,5*S*,8*S*,8a*S*)-2,4,4a,5,6,7,8,8$^{a}$-Octahydro-8a-hydroxy-3,4a,5-trimethyl-2-oxonaphtho(2,3-b) furan-4,8-diyl diacetate. | *L. sagitta* | root | [40] |
| **1-60** | No Name | *L. veitchiana* | root | [18] |
| **1-61** | 3β-Acetyloxy-6α-hydroxyligularenolide | *L. przewalskii* | root | [41] |
| **1-62** | 6β-Angeloyloxy-2-oxoeremophil-1(10),7(11),8-triene-12,8-olide | *L. virgaurea* | root | [42] |
| **1-63** | 3β-Acetoxy-10β-hydroxy-6β-methoxy-eremophil-7(11),8(9)-dien-8,12-olide | *L. hodgsonii* | root and aerial part | [36] |
| **1-64** | 6β-(2*E*-Methylbutyryloxy)eremophil-3,7(11),8-trien-8,12-olide-15-oic acid methyl ester | *L. lapathifolia* | root | [5] |
| **1-65** | (3β,6β,10β)-3-Acetyl-6,10-dihydroxy-eremophila-7(11),8-dieno-12,8-lactone | *L. fischeri* | root | [23] |
| **1-66** | (3β,6β,10α)-3-Acetyl-6,10-dihydroxy-eremophila-7(11),8-dieno-12,8-lactone | *L. fischeri* | root | [23] |

| | | | | |
|---|---|---|---|---|
| **1-67** | 6α,10α-Dihydroxy-1-oxoeremophila-7(11),8(9)-dien-8,12-olide | *L. virgaurea* spp. *oligocephala* | whole plant | [43] |
| **1-68** | 6β,10α-Dihydroxy-1-oxoeremophila-7(11),8(9)-dien-8,12-olide | *L. virgaurea* spp. *oligocephala* | whole plant | [43] |
| **1-69** | 10α-Hydroxy-1-oxoeremophila-7(11),8(9)-dien-8,12-olide | *L. virgaurea* spp. *oligocephala* | whole plant | [43] |
| **1-70**[a] | Virgauride | *L. virgaurea* | root | [44] |
| **1-71**[a] | Virgauride | *L. virgaurea* | root | [44] |
| **1-72** | 6β-Acetyl-2-oxoeremophila-1(10),7(11),8(9)-trien-12,8-olide | *L. virgaurea* spp. *oligocephala* | whole plant | [31] |
| **1-73** | 6β,10β-Dihydroxyeremophila-7(11),8(9)-dien-12,8-olide | *L. virgaurea* spp. *oligocephala* | whole plant | [45] |
| **1-74** | Eremofarfugin C | *L. kanaitzensis* | root | [24] |
| **1-75** | 6β-Hydroxy-3β-(2'-methylacryloyl)oxy-11*βH*-eremophil-7-en-12,8-olide | *L. virgaurea* | root | [42] |
| **1-76** | Eremofarfugin D | *L. vellerea* | root | [46] |
| **1-77** | Eremofarfugin E | *L. vellerea* | root | [46] |
| **1-78** | 3β-(2'-Methylbutanoyloxy)-8*βH*-eremophil-7(11)-ene-12,8α(14,6α)-diolide | *L. tongolensis* | root | [47] |
| **1-79** | 3β-Angeloyloxy-8βH-eremophil-7(11)-ene-12,8α(14β,6α)-dioxide | *L. lapathifolia* | root and rhizome | [48] |
| **1-80** | (1*R*,4*S*,5*S*,6*R*,8*S*,10*R*)-1-Acetoxyeremophil-7(11)-en-6,15;8,12-diolide | *L. hodgsonii* | root and rhizome | [37] |
| **1-81** | 8β-Methoxyeremophil-7(11)-en-6α,15;8α,12-diolie | *L. przewalskii* | root | [21] |
| **1-82** | 10β-Hydroxy-8β,9β-epoxyeremophil-7(11)-en-6α,15;8α,12-diolide | *L. przewalskii* | root | [21] |
| **1-83** | 3β-Angeloyloxy-8*βH*-hydroxyeremophil-7(11)-ene-12,8α(14β,6α)-dioxide | *L. lapathifolia* | root and rhizome | [48] |
| **1-84** | (1*R*,4*S*,5*S*,6*R*,8*S*,10*R*)-1-Acetoxy-8β-hydroxyeremophil-7(11)-en-6,15;8,12-diolide | *L. hodgsonii* | root and rhizome | [37] |
| **1-85** | (4*S*,5*S*,6*R*,8*R*,9*S*,10*S*)-8-Hydroxy-9-(angeloyloxy)eremophil-7(11)-en-6,15;8,12-diolide | *L. hodgsonii* | root and rhizome | [37] |
| **1-86** | 3β-Angeloyloxy-8*βH*-methoxyeremophil-7(11)-ene-12,8α(14β,6α)-dioxide | *L. lapathifolia* | root and rhizome | [48] |
| **1-87** | 3β-Angeloyloxy-8*βH*-ethoxyeremophil-7(11)-ene-12,8α(14β,6α)-dioxide | *L. lapathifolia* | root and rhizome | [48] |
| **1-88** | 7α,8α-Epoxy-eremophil-an-12β, 8β(14β,6α)-diolide | *L. intermedia* | rhizome | [49] |
| **1-89** | 8α-Hydroxyeremophil-7(11)-ene-12,8β(14β,6α)-diolide | *L. intermedia* | rhizome | [50] |
| **1-90** | Eremophil-7(8)-en-12,8(14β, 6α)-diolide | *L. intermedia* | rhizome | [49] |
| **1-91** | Eremophil-8(9),7(11)-dien-6α,15;8,12-diolide | *L. przewalskii* | root | [21] |
| **1-92** | 10β-Hydroxyeremophil-8(9),7(11)-dien-6α,15;8,12-diolide | *L. przewalskii* | root | [21] |
| **1-93** | (4*S*,5*S*,6*R*,10*R*)-10-Hydroxyeremophil-7(11),8(9)-diene-6,15;8,12-diolide | *L. hodgsonii* | root and rhizome | [37] |
| **1-94** | 3β-Angeloyloxy-10β-hydroxyeremophil-8(9),7(11)-diene-12,8(14β,6α)-dioxide | *L. lapathifolia* | root and rhizome | [48] |
| **1-95** | 8*βH*-Eremophil-3, 7(11)-dien-12,8α(14,6α)-diolide | *L. tongolensis* | root | [47] |
| **1-96** | 8β-Hydroxyeremophil-3,7(11)-diene-8α,12(6α,15)-diolide | *L. lapathifolia* | root | [5] |
| **1-97** | 8β-Methoxyeremophil-3,7(11)-diene-8R,12(6R,15)-diolide | *L. lapathifolia* | root | [5] |
| **1-98** | 8β-Ethoxyeremophil-3,7(11)-diene-8α,12(6α,15)-diolide | *L. lapathifolia* | root | [5] |
| **1-99** | 8α-Hydroxy-eremophil-3,7(11)-dien-12,8β(14,6α)-diolide | *L. atroviolacea* | root | [51] |
| **1-100** | 1α-Acetoxyfuranoeremophilan-15,6α-olide | *L. dictyoneura* | root | [52] |
| **1-101** | 3β-Angeloyloxyeremophilan-7,11-diene-14β,6α-olide | *L. lapathifolia* | root and rhizome | [48] |
| **1-102** | 3β-Angeloyloxy-8,12-epoxy-12α-hydroxy-8β-methoxyeremophil-7(11)-en-14β,6α-olide | *L. lapathifolia* | root and rhizome | [48] |
| **1-103** | Ligumacrophyllatin | *L. macrophylla* | root | [53] |
| **1-104** | 9-Oxoplatyphyllide | *L. fischeri* | root | [54] |
| **1-105** | 6α,9-Dihydroxy-14β-carboxyfuranoeremophil-9(10)-ene | *L. intermedia* | rhizome | [49] |
| **1-106** | 1,3-Dimethoxy-4,6,11-trimethylnapbthofuran | *L. przewalskii* | root | [55] |
| **1-107** | 6β-Angeloyloxy-furanoligularenone | *L. pleurocaulis* | root and rhizome | [56] |
| **1-108** | 6β-Acetoxyfuranoeremophilan-10β-ol | *L. kanaitzensis* | root | [24] |
| **1-109** | Subspicatin A | *L. subspicata* | root | [34] |
| **1-110** | Subspicatin B | *L. subspicata* | root | [34] |
| **1-111** | Subspicatin C | *L. subspicata* | root | [34] |
| **1-112** | 6β-Angeloyloxyfuranoeremophil-1(10)-en-2-one | *L. virgaurea* | root | [42] |
| **1-113** | 6β-Acetoxyfuranoeremophil-1(10)-en-2-one | *L. virgaurea* | root | [42] |
| **1-114** | 6β-Hydroxyfuranoeremophil-1(10)-en-2-one | *L. virgaurea* | root | [42] |
| **1-115** | (4*S*,4a*S*,5*S*,8*R*,8a*S*)-4,4a,5,6,7,8,8a,9-Octahydro-8,8a-dihydroxy-3,4a,5-trimethylnaphtho(2,3-b)furan-4-yl(2-(hydroxymethyl)prop-2-enoate | *L. sagitta* | root | [40] |
| **1-116** | 1α-Chloro-6β-isobutyroxy-9-oxo-10β-hydroxy-furanoeremophilane | *L. atroviolacea* | root | [57] |
| **1-117** | 6β-Sarracinoyloxy-1β,10β-epoxy-furanoeremophilane | *L. macrophylla* | root and rhizome | [58] |
| **1-118** | 6β-(*Z*-4'-Hydroxy-2-methyl-2-butenoyl)oxyfuranoeremophil-1(10)-en-2-one | *L. virgaurea* | root | [42] |
| **1-119** | 6α-Angeloyloxy-10*βH*-furanoeremophil-1-one | *L. macrophylla* | root and rhizome | [58] |
| **1-120** | 1α-Hydroxy-9-deoxycacalol | *L. macrophylla* | root and rhizome | [58] |
| **1-121** | 3β-Acetoxy-6β-(angeloyloxy)furanoeremophilan-10β-ol | *L. dictyoneura* | root | [52] |
| **1-122** | Franchetianone B | *L. franchetiana* | root | [59] |

| | | | | |
|---|---|---|---|---|
| **1-123** | 6*β*-(2-(Hydroxymethyl)prop-2-enoyloxy)furanoeremophil-1(10)-ene | *L. dictyoneura* | root | [52] |
| **1-124** | 1*β*,10*α*-Dihydroxy-6*β*-((2-methylpropyl)oxy)furanoeremophil-9-one | *L. virgaurea* spp. *oligocephala* | whole plant | [1] |
| **1-125** | 3*β*-Acetoxy-6*β*-(2-methylbutyryloxy)furanoeremophilan-10*β*-ol | *L. oligonema* | root | [60] |
| **1-126** | 3*β*-Acetoxy-6*β*-isobutyryloxyfuranoeremophilan-10*β*-ol | *L. oligonema* | root | [60] |
| **1-127** | Furanoeremophil-1(10)-en-6*α*-ol | *L. anoleuca* | root | [61] |
| **1-128** | Benzofuranoeremophil-2-ene | *L. sagitta* | rhizome | [62] |
| **1-129** | 1,3-Dimethoxy-4,6-dimethylnaphthofuran | *L. veitchiana* | root | [4] |
| **1-130** | Eremoligularin | *L. muliensis* | root | [63] |
| **1-131** | Subspicatolide | *L. subspicata* | root | [34] |
| **1-132** | Franchetianone A | *L. franchetiana* | root | [59] |
| **1-133** | 1*β*-Hydroxy-6*α*-isobutyryloxy-9-noreremophil-7(11),8(10)-dien-8(12)-olide | *L. fischeri* | root | [54] |
| **1-134** | 1*β*-Acetoxy-6*α*-isobutyryloxy-9-noreremophil-7(11),8(10)-dien-8(12)-olide | *L. fischeri* | root | [54] |
| **1-135** | Ligulactone A | *L. fischeri* | root | [14] |
| **1-136** | Ligulactone B | *L. fischeri* | root | [14] |
| **1-137** | Eremophila-1(10),7(11),8-triene-12,8-lactam | *L. fischeri* | root | [3] |
| **1-138** | No Name | *L. veitchiana* | root | [18] |
| **1-139** | 1*β*,11-Dihydroxy-eremophil-9-ene | *L. fischeri* | whole plant | [64] |
| **1-140** | Ligudicin A | *L. dictyoneura* | root and rhizome | [65] |
| **1-141** | 11-Hydroxy-eremophil 1(10)-en-2,9-dione | *L. fischeri* | whole plant | [64] |
| **1-142** | 7*α*-Hydroxy-9-en-1,8-dioxo-6,7-dihydroxyfuranoeremophilane | *L. veitchiana* | whole plant | [27] |
| **1-143** | 8-((*β*-D-Glucopyranosyl)oxy)eremophila-1(10),8,11-trien-2-one | *L. virgaurea* spp. *oligocephala* | whole plant | [66] |
| **1-144** | (7*α*,9*α*,10*α*)-9,10-Epoxy-eremophilan-11-ol | *L. veitchiana* | rhizome | [67] |
| **1-145** | 3*α*-Tigloyloxyeremophila-9,11-dien-8-one | *L. kanaitzensis* | root | [24] |
| **1-146** | 3*α*-Propionyloxy-7*βH*-eremophila-9,11-dien-8-one | *L. kanaitzensis* | root | [24] |
| **1-147** | (4a*S*,5*S*,8*R*)-5,6,7,8-Tetrahydro-8-hydroxy-3-(1-hydroxypropan-2-yl)-4a,5-dimethyl-naphthalen-2(4a*H*)-one | *L. sagitta* | root | [40] |
| **1-148** | 1*β*-Acetoxy-11(*S*)-8-oxoeremophil-6,9-dien-12-al | *L. sagitta* | root | [68] |
| **1-149** | 1*β*-Acetoxy-11(*R*)-8-oxoeremophil-6,9-dien-12-al | *L. sagitta* | root | [68] |
| **1-150** | 1*β*,12-Diacetoxy-6,9,12*E*-trien-8-oxoeremophilane | *L. sagitta* | root | [68] |
| **1-151** | 3*β*-Angeloyloxy-8-oxoeremophil-6(7)-ene-12,15-dioic acid methyl ester | *L. lapathifolia* | root | [5] |
| **1-152** | Eremophil-6-en-11-ol | *L. veitchiana* | rhizome | [67] |
| **1-153** | 11(R)-8-Oxoeremophil-6(7)-en-dimethyl-12,15-dioate | *L. przewalskii* | root | [21] |
| **1-154** | 11(S)-8-Oxoeremophil-6(7)-en-dimethyl-12,15-dioate | *L. przewalskii* | root | [21] |
| **1-155** | 3*β*-Acetyloxy-11-methoxy-8-oxoeremophila-6,9-dien-12-oic acid | *L. przewalskii* | root | [41] |
| **1-156** | Kanaitzensol | *L. kanaitzensis* | root | [24] |
| **1-157** | 1*β*-Hydroxy-11(*R*,*S*)-8-oxoeremophil-6,9-dien-12-al | *L. macrophylla* | root and rhizome | [58] |
| **1-158** | 1*β*,10*β*-Epoxy-7(11)-en-6*α*-hydroxy-8-oxo-eremophil-12-oic acid | *L. sagitta* | root | [69] |
| **1-159** | 3*β*-Acetyloxy-11-(2'-methylbutanoyloxy)-8-oxoeremophila-6,9-dien-12-oic acid | *L. przewalskii* | root | [41] |
| **1-160** | (1*α*)-1-Hydroxy-8-oxo-eremophila-6,9-dien-12-oic acid | *L. virgaurea* spp. *oligocephala* | whole plant | [66] |
| **1-161** | 1*β*-Angeloyloxy-8-oxoeremophil-6,9-dien-12-oic acid Me ester | *L. myriocephala* | whole plant | [30] |
| **1-162** | 3*β*-Acetoxy-8-oxoeremophil-6(7),9(10)-dien-12-oic methyl ester | *L. hodgsonii* | root and aerial part | [36] |
| **1-163** | 2-Acetyl-8a-methyl-2-(2-methyl-but-2-enoyloxy)-6-oxo-1,2,3,4,4a,5,6,8a-octahydro-naphthalene-1-carboxylic acid | *L. lapathifolia* | root and rhizome | [70] |
| **1-164** | 1*β*-Hydroxy-6(7),9(10)-dien-8-oxo-eremophil-12-oic acid | *L. veitchiana* | whole plant | [28] |
| **1-165** | (1*R*,4*S*,4a*R*)-6-Acetyl-1,2,3,4,4a,5-hexahydro-4,4$^a$-dimethylnaphthalene-1,7-diyl diacetate | *L. sagitta* | root | [40] |
| **1-166** | (4a*S*,5*S*,8*R*)-5,6,7,8-Tetrahydro-3,8-dihydroxy-4a,5-dimethylnaphthalen-2(4a*H*)-one | *L. sagitta* | root | [40] |
| **1-167** | (2*R*,5*R*,8*S*,8a*R*)-1,2,3,5,6,7,8,8$^a$-Octahydro-5-hydroxy-8,8a-dimethyl-3-oxonaphthalen-2-yl acetate | *L. sagitta* | root | [40] |
| **1-168** | 3*β*-((2-Methylacryloyl)oxy)-8-oxo-12-noreremophil-6-en-11-one | *L. virgaurea* | root | [17] |
| **1-169** | 9*β*-Hydroxy-8-oxo-12-noreremophil-6-en-11-one | *L. virgaurea* | root | [17] |
| **1-170** | 3*β*-(3-Methylbutanoyloxy)-11-noroxoeremophila-6(7),9(10)-diene-8,11-dione | *L. japonica* | root | [16] |
| **1-171** | 3*β*-Acetyloxy-6*α*,7*α*-epoxy-11-noreremophil-9(10)-ene-8,11-dione | *L. japonica* | root | [16] |
| **1-172** | 1*β*-Hydroxy-6,9-dien-8-oxoeremophil-11-nor-11-ketone | *L. veitchiana* | root | [68] |
| **1-173** | 1*β*-Acetoxy-6*α*,7*α*-epoxy-9-en-8-oxoeremophil-11-nor-11-ketone | *L. veitchiana* | root | [68] |
| **1-174** | (1Z)-1-((5*R*,8*S*,8a*R*)-5-Acetoxy-6,7,8,8a-tetrahydro-8,8a-dimethyl-3-oxonaphthalen-2(1*H*)-ylidene) ethyl acetate | *L. sagitta* | root | [40] |
| **1-175** | 1*β*-Acetoxy-6,9-dien-8-oxoeremophil-11-nor-11-ketone | *L. sagitta* | root | [68] |
| **1-176** | 3*β*-Hydroxy-11-noreremophila-6(7),9(10)-diene-8,11-dione | *L. japonica* | root | [16] |
| **1-177** | Ligudentatin A | *L. dentata* | root | [71] |
| **1-178** | Ligudentatin B | *L. dentata* | root | [71] |
| **1-179** | 8*α*-Hydroxyligudentatol | *L. dentata* | root | [72] |
| **1-180** | 8*α*-Hydroxyligujapone | *L. dentata* | root | [72] |
| **1-181** | 7-Acetyl-1-hydroxy-4-methylene-1,2,3,4-tetrahydronaphthalene | *L. duciformis* | root | [73] |
| **1-182** | 1*β*-Hydroxy-6(7),9(10)-dien-8-oxo-12-nor-11-hydroxy-eremophiladiene | *L. veitchiana* | whole plant | [28] |
| **1-183** | (4a*S*,5*S*)-5,6,7,8-Tetrahydro-3-hydroxy-4a,5-dimethylnaphthalen-2(4a*H*)-one | *L. fischeri* | root | [74] |
| **1-184** | 3*β*-Acetyloxy-7-hydroxynoreremophila-6,9-dien-8-one | *L. przewalskii* | root | [41] |
| **1-185** | 8*β*-Hydroxy-2-dehydroxyliguhodgsonal | *L. przewalskii* | root | [41] |
| **1-186** | Liguladentanorol | *L. dentata* | root | [72] |
| **1-187** | 10*β*H-6*β*-Acetoxy-7*β*,11*β*-epoxyeremophilan-8-one | *L.lamarum* | root | [75] |
| **1-188** | 10*β*H-6*β*-Acetoxy-7*a*,11*a*-epoxyeremophilan-8-one | *L.lamarum* | root | [75] |
| **1-189** | 10*β*H-8*a*,11-Epidioxyeremophil-6-en-8*β*-ol | *L.lamarum* | root | [75] |

| | | | | |
|---|---|---|---|---|
| **1-190** | 12-Hydroxy-6-oxo-6,7-secobakk-7(11)-en-8,12-olide | *L. subspicata* | root | [75] |
| **1-191** | Ligudentatol | *L. dentata* | rhizome | [76] |
| **1-192** | Ligularate | *L. fischeri* | root | [54] |
| **1-193** | 2-Acetyl-3a-methyl-5-(2-methyl-but-2-enoyloxy)-3a,4,5,6,7,7a–hexahydro-1*H*-indene-4-carboxylic acid | *L. lapathifolia* | root and rhizome | [70] |
| **1-194** | 2-Acetyl-3a*β*-methyl-3a,6,7,7a*β*-tetrahydro-1*H*-inden-4-oic acid methyl ester | *L. lapathifolia* | root | [5] |
| **1-195** | 1-((3a*R*,4*S*,7a*R*)-3a,4,5,6,7a-Hexahydro-3a,4-dimethyl-1*H*-inden-2-yl)ethanone | *L. virgaurea* | root | [42] |
| **1-196** | 2-Acetyl-3*α*,*β*-methyl-3*α*,4,5,6,7,7a-hexahydroinden-4*β*-carboxylic methyl ester | *L. przewalskii* | root | [21] |
| **1-197** | (3a*R*,4*R*,5*S*,7a*S*)-2-Acetyl-3a,4,5,6,7,7a-hexahydro-7a-hydroxy-1*H*-inden-5-yl acetate | *L. fischeri* | root | [23] |
| **1-198** | 5*β*-Angeloyloxy-3a,4,5,6,7,7a-hexahydro-3a*β*-methyl-1*H*-indene-2,4*β*-dioic acid methyl ester | *L. virgaurea* | root | [35] |
| **1-199** | Ligulasagitin A | *L. sagitta* | root | [77] |
| **1-200** | Ligulasagitin B | *L. sagitta* | root | [77] |
| **1-201** | Ligulasagitin C | *L. sagitta* | root | [77] |
| **1-202** | Ligulaverin A | *L. veitchiana* | whole plant | [78] |
| **1-203** | Ligulaverin B | *L. veitchiana* | whole plant | [78] |
| **1-204** | Ligulaverin C | *L. veitchiana* | whole plant | [78] |
| **1-205** | Ligulaverin D | *L. veitchiana* | whole plant | [78] |
| **1-206** | Ligulaverin E | *L. veitchiana* | whole plant | [78] |
| **1-207** | No Name | *L. sagitta* | root | [2] |
| **1-208** | No Name | *L. sagitta* | root | [2] |
| **1-209** | No Name | *L. sagitta* | root | [2] |
| **1-210** | No Name | *L. sagitta* | root | [2] |
| **1-211** | Altaicalarin C | *L. altaica* | root and rhizome | [79] |
| **1-212** | (1*β*,2*β*,3*β*,4*α*,6*β*)-Bisabol-7(14)-ene-1,2,3,4,8,10,11-heptol 2,10-diangelate | *L. cymbulifera* | root | [80] |
| **1-213** | (1*β*,2*β*,3*β*,4*β*,6*β*)-3,4-Epoxybisabol-7(14)-ene-1,2,8,10,11-pentol 2,10-diangelate | *L. cymbulifera* | root | [80] |
| **1-214** | 2*β*,8-Bisangeloyloxy-3*β*,4*β*,10,11-bisepoxybisabol-7(14)-en-1*β*-ol | *L. lankongensis* | root | [81] |
| **1-215** | 2*β*,8-Bisangeloyloxy-4*α*-chloro-10,11-epoxybisabol-7(14)-ene-1*β*,3*β*-diol | *L. lankongensis* | root | [81] |
| **1-216** | (1*β*,2*β*,3*β*,4*α*,6*β*)-Bisabol-7(14)-ene-1,2,3,4,8,10,11-heptol2,10-diangelate-4-((3*S*,5*S*,6*S*)-tetrahydroclivonecate) | *L. cymbulifera* | root | [80] |
| **1-217** | 10,11-Epoxy-1*β*-hydroxy-2*β*,4*α*,8-triangeloyloxybisabol-7(14)-ene | *L. cymbulifera* | root | [82] |
| **1-218** | 2*β*,8-Diangeloyloxy-3*α*,4*α*,10,11-diepoxy-1*α*-hydroxybisabol-7(14)-ene | *L. cymbulifera* | root | [82] |
| **1-219** | 2*α*,8-Diangeloyloxy-3*β*,4*β*,10,11-diepoxy-1*α*-hydroxybisabol-7(14)-ene | *L. cymbulifera* | root | [82] |
| **1-220** | (1*α*,2*α*,3*β*,5*α*,6*β*)-2,5,8-Tris(angeloyloxy)-10,11-epoxy-1,3-dihydroxybisabol-7(14)-en-4-one | *L. dentata* | root | [83] |
| **1-221** | (1*α*,2*β*,3*β*,4*R**,5*α*,6*α*)-3,6,9-Tris((angeloyl)oxy)bisabol-10(15)-ene-2,4,5,7,11-pentol | *L. lankongensis* | root | [84] |
| **1-222** | (1*α*,2*β*,3*β*,4*R**,5*α*,6*α*)-3,6,7-Tris((angeloyl)oxy)bisabol-10(15)-ene-2,4,5,9,11-pentol | *L. lankongensis* | root | [84] |
| **1-223** | Altaicalarin D | *L. altaica* | root and rhizome | [79] |
| **1-224** | Ligudentatone A | *L. dentata* | root | [85] |
| **1-225** | Ligudentatone B | *L. dentata* | root | [85] |
| **1-226** | 4*α*-Chloro-1*β*,8-diangeloyloxy-10,11-epoxy-2*β*-hydroxybisabol-7(14)-ene | *L. cymbulifera* | root | [82] |
| **1-227** | 1*β*,5*α*,8-Trisangeloyloxy-3*β*,4*β*,10,11-bisepoxybisabol-7(14)-en-2*β*-ol | *L. lankongensis* | root | [81] |
| **1-228** | 3*β*,4*β*,10,11-Diepoxy-1*β*,2*β*,8-triange-loyloxybisabol-7(14)-ene | *L. cymbulifera* | root | [82] |
| **1-229** | 1*β*,8-Diangeloyloxy-3*β*,4*β*,10,11-diepoxybisabol-7(14)-ene | *L. cymbulifera* | root | [82] |
| **1-230** | 1*β*,8-Diangeloyloxy-3*β*,4*β*-epoxy-2*β*,10,11-trihydroxybisabol-7(14)-ene | *L. cymbulifera* | root | [82] |
| **1-231** | 1*α*,8-Diangeloyloxy-10,11-dihydroxy-3*β*,4*β*-epoxybisabol-7(14)-en-2-one | *L. cymbulifera* | root | [82] |
| **1-232** | (1*S*,2*R*,6*S*,9*R*)-2,7-Bis(angeloyloxy)-6,9-epoxy-11-hydroxy-6-methoxybisabola-3,10(15)-dien-5-one | *L. dentata* | root | [86] |
| **1-233** | (1*α*,2*α*,3*β*,5*α*,6*β*)-1,5,8-Tris(angeloyloxy)-10,11-epoxy-2,3-dihydroxybisabol-7(14)-en-4-one | *L. dentata* | root | [83] |
| **1-234** | (1*α*,2*β*,3*β*,5*α*,6*β*)-1,8-Bis(angeloyloxy)-2,3-epoxy-5,10-dihydroxy-11-metho-xybisabol-7(14)-en-4-one | *L. dentata* | root | [83] |
| **1-235** | 8-Angeloyloxy-3*β*,4*β*,10,11-bisepoxy-1*β*-(2–methylbutyryloxy)bisabol-7(14)-en-2*β*-ol | *L. lankongensis* | root | [81] |
| **1-236** | (1*E*,5*R*)-3-Acetoxy-9-angeloyloxy-5-hydroxybisabola-3,1(10),7(11)-trien-2-one | *L. dentata* | root | [86] |
| **1-237** | 9-Angeloyloxy-11-methoxybisabola-1,3,5,10(15)-tetraene-5,6,7-triol | *L. dentata* | root | [86] |
| **1-238** | 9*α*-Angeloyloxy-7*β*,10*β*-epoxy-11-methoxybisabola-1,3,5-triene-5,6-diol | *L. dentata* | root | [86] |
| **1-239** | (8*β*,10*α*)-8-Angeloyloxy-5,10-epoxybisabola-1,3,5,7(14)-tetraene-2,4,11-triol | *L. dentata* | root | [83] |
| **1-240** | (8*β*,10*α*)-8-Angeloyloxy-5,10-epoxythiazolo(5,4-a)bisabola-1,3,5,7(14)-tetraene-4,11-diol | *L. dentata* | root | [83] |
| **1-241** | Altaicalarin A | *L. altaica* | root and rhizome | [79] |
| **1-242** | Altaicalarin B | *L. altaica* | root and rhizome | [79] |
| **1-243** | (1*R*,5*S*,6*R*,7*S*,8*R*,9*S*,11*S*)-4-Acetoxy-9-((4-acetoxy-4-methylsenecioyl)oxy)-8-((2-methylbutanoyl)oxy)-11,12-epoxyoplop-10(14)-en-3-one | *L. narynensis* | root | [87] |
| **1-244** | (1*S*,3a*R*,5*S*,6*R*,7*S*,7a*R*)-1-(1-Acetoxyethyl)octahydro-4-methylidene-7-((2*S*)-2-methyl-oxiran-2-yl)-6-(3-methylpentanoyl)oxy)-2-oxo-1*H*-inden-5-yl-(2*E*)-4-acetoxy-3-methylpent-2-enoate | *L. narynensis* | root | [87] |
| **1-245** | (1*S*,2*R*,3*S*,3a*R*,5*S*,6*R*,7*S*,7a*S*)-2-Acetoxy-1-(1-acetoxyethyl)octahydro-3,6-bis((2-methylbutanoyl)-oxy)-4-methylidene-7-((2*S*)-2-methyloxiran-2-yl)-1*H*-inden-5-yl(2*E*)-4-hydroxy-3-methylpent-2-enoate | *L. narynensis* | root | [87] |
| **1-246** | (1*S*,2*R*,3*S*,3a*R*,5*S*,6*R*,7*S*,7a*S*)-2-Acetoxy-1-(1-acetoxyethyl)-octahydro-3,6-bis((2-methylbutanoyl)-oxy)-4-methylidene-7-((2*S*)-2-methyloxiran-2-yl)-1*H*-inden-5-yl(2*E*)-3-methylpent-2-enoate | *L. narynensis* | root | [87] |
| **1-247** | 3*β*,4-Diacetoxy-9*α*-(4-acetoxy-4-methyl-senecioyloxy)-2*β*,8*α*-di(2-methylbutyryl-oxy)-11*α*,12-epoxyoplop-10,(14)-ene | *L. narynensis* | root | [88] |
| **1-248** | 3*β*,4-Diacetoxy-8*α*-(2-methylbutyryloxy)-9*α*-(4-methylsenecioyloxy)-11*α*,12-epoxyoplop-10,(14)-ene | *L. narynensis* | root | [88] |
| **1-249** | (5*β*,9*β*)-Guaia-6,10(14)-dien-9-ol | *L. macrophylla* | root and rhizome | [89] |

| | | | | |
|---|---|---|---|---|
| **1-250** | 2α-Hydroxy-1βH,7αH,10αH-guai-4,11(12)-dien-3-one | *L. narynensis* | root | [88] |
| **1-251** | Liguducin A | *L. duciformis* | whole plant | [90] |
| **1-252** | Furanomexicanane-9-ene-8-one | *L. virgaurea* | root | [44] |
| **1-253** | 9β,10β-Epoxy-furanomexicanane-8-one | *L. virgaurea* | root | [44] |
| **1-254** | 4α,8β,9α-Trihydroxy-5αH-7(11)-eudesmen-12,8α-olide | *L. platyglossa* | root and rhizome | [38] |
| **1-255** | Eudesma-4,11-diene-1β,15-diol | *L. dentata* | root | [86] |
| **1-256** | Liguducin B | *L. duciformis* | whole plant | [90] |
| **1-257** | (+)-Intermedeol | *L. fischeri* var. *spiciformis* | leaves | [25] |
| **1-258** | Ligucyperonol | *L. dentata* | rhizome | [76] |
| **1-259** | 2-(3-Pentenyl)-3,7-dimethylbenzofuran-1,4-dione | *L. virgaurea* | rhizome | [91] |
| **1-260** | 1-Hydroxy-2-(3-pentenyl)-3,7-dimethylbenzofuran | *L. virgaurea* | rhizome | [91] |
| **1-261** | 1-Methoxy-2-(3–pentenyl)-3,7-dimethylbenzofuran | *L. virgaurea* | rhizome | [91] |
| **1-262** | Ligulolide A | *L. virgaurea* spp. *oligocephala* | whole plant | [9] |
| **1-263** | (6*S*) Ligulolide C | *L. virgaurea* spp. *oligocephala* | whole plant | [92] |
| **1-264** | (6*R*) Ligulolide C | *L. virgaurea* spp. *oligocephala* | whole plant | [92] |
| **1-265** | Ligupersin A | *L. persica* | root | [93] |
| **1-266** | Ligupersin B | *L. persica* | root | [93] |
| **1-267** | Virgauronin | *L. virgaurea* | root | [94] |
| **1-268** | Bieremoligularolide | *L. muliensis* | root | [63] |
| **1-269** | 8β-(Eremophil-3',7'(11')-dien-12',8'α;15',6'α-diolide)-eremophil-3,7(11)-dien-12,8α;15,6α-diolide | *L. atroviolacea* | root | [57] |
| **1-270** | Fischelactone | *L. fischeri* | root | [3] |
| **1-271** | 8,8'-bi-3β-Angeloyloxy-eremophil-7(11)-en-12,8α(14β,6α)-diolide | *L. lapathifolia* | root and rhizome | [95] |
| **1-272** | Biligulaplenolide | *L. platyglossa* | root and rhizome | [38] |
| **1-273** | Virgaurol A | *L. virgaurea* | root | [96] |
| **1-274** | Virgaurol B | *L. virgaurea* | root | [96] |
| **1-275** | Ligulasagitin D | *L. sagitta* | root | [77] |
| **1-276** | Virgaurin A | *L. virgaurea* | root | [44] |
| **1-277** | Ligularin A | *L. virgaurea* spp. *oligocephala* | whole plant | [1] |
| **1-278** | 2-{((5*S*)-5,6,7,8-Tetrahydro-9-hydroxy-3,5-dimethylnaphtho(2,3-b)furan-4-yl)methyl}-3,5-dimethyl-6-((3*E*)-pent-3-en-1-yl)-1-benzofuran-4,7-dione | *L. virgaurea* | rhizome | [97] |
| **1-279** | (5*S*)-5,6,7,7a,7b,12b-Hexahydro-3,4,5,11,12b-pentamethy-10-((3*E*)-pent-3-en-1-yl)-furo(3",2":6',7')-naphtho(1',8': 4,5,6)pyrano(3,2-b)benzofuran-9-ol | *L. virgaurea* | rhizome | [97] |
| **1-280** | Ligulolide D | *L. virgaurea* spp. *oligocephala* | whole plant | [1] |
| **1-281** | Ligulolide B | *L. virgaurea* spp. *oligocephala* | whole plant | [31] |
| **1-282** | Biliguhodgsonolide | *L. hodgsonii* | root and rhizome | [98] |
| **1-283** | Ligulamulienin A | *L. muliensis* | rhizome | [99] |
| **1-284** | Ligulamulienin B | *L. muliensis* | rhizome | [99] |
| **1-285** | Virgaurol D | *L. virgaurea* | root | [96] |
| **1-286** | Virgaurol C | *L. virgaurea* | root | [96] |
| **1-287** | Ligulasagitin E | *L. sagitta* | root | [77] |
| **1-288** | Ligulatrovine A | *L. atroviolacea* | root | [57] |
| **1-289** | Ligumacrophyllal | *L. macrophylla* | root | [53] |

[a]Compounds **1-70** and **1-71** were obtained as a pair of epimers at C-6, and both of them were named virgauride.

**Table 2. Monoterpenoids and diterpenoids from the genus *Ligularia***

| No. | compound class and name | plant source | part of plant | Ref. |
|---|---|---|---|---|
| | ***Monoterpenoids*** | | | |
| **2-1** | rel-(1*R*,2*R*,3*R*,4*S*,5*S*)-p-Menthane-1,2,3,5-tetrol | *L. muliensis* | root | [15] |
| **2-2** | 1α,2β,3α,6α-Tetrahydroxy-p-menthane | *L. narynensis* | root | [88] |
| | ***Diterpenoids*** | | | |
| **2-3** | Sagittolactone | *L. sagitta* | rhizome | [62] |
| **2-4** | Spiciformisin A | *L. fischeri* var. *spiciformis* | leaves | [100] |
| **2-5** | Spiciformisin B | *L. fischeri* var. *spiciformis* | leaves | [100] |

**Table 3. Triterpenoids from the genus *Ligularia***

| No. | compound name | plant source | part of plant | Ref. |
|---|---|---|---|---|
| **3-1** | 7*β*,16*β*,28-Triacetoxyolean-12-en-3-one | *L. sagitta* | root | [40] |
| **3-2** | 3-oxo-16*β*-Hydroxy-olean-12-ene-28-al | *L. odontomanes* | whole plant | [101] |
| **3-3** | Liguveitoside B | *L. veitchiana* | root and rhizome | [102] |
| **3-4** | Liguveitoside A | *L. veitchiana* | whole plant | [103] |
| **3-5** | 3,4-Seco-olean-12-en-4-ol-3,28-dioic acid | *L. intermedia* | root | [104] |
| **3-6** | A-homo-3a-oxa-olean-12-en-3-one-28-oic acid | *L. intermedia* | root | [104] |
| **3-7** | 2*α*,3*β*,19*α*-trihydroxy-28-norurs-12-ene | *L. tongolensis* | root | [47] |
| **3-8** | 2*α*,3*α*,19*α*-trihydroxy-28-norurs-12-ene | *L. tongolensis* | root | [47] |
| **3-9** | Monocyclosqualene | *L. fischeri* var. *spiciformis* | leaves | [100] |
| **3-10** | 24-Chlorocycloart-25-en-3*β*-ol | *L. stenocephala* | root and leaves | [105] |

**Table 4. Other chemical constituents from the genus *Ligularia***

| No. | compound class and name | plant source | part of plant | Ref. |
|---|---|---|---|---|
| | ***Steroids*** | | | |
| **4-1** | 3*β*,7*α*,22-Trihydoxystigmast-5-ene | *L. dolichobotrys* | whole plant | [39] |
| | ***Alkaloids*** | | | |
| **4-2** | *O*-Acetylyamataimine | *L. tsangchanensis* | root | [106] |
| **4-3** | *O*-Acetylyamataimine N-oxide | *L. tsangchanensis* | root | [106] |
| **4-4** | Clivorine | *L. hodgsonii* | whole plant | [107] |
| **4-5** | Ligularine | *L. hodgsonii* | whole plant | [107] |
| **4-6** | Lankongensisine A | *L. lankongensis* | root | [108] |
| **4-7** | Lankongensisine B | *L. lankongensis* | root | [108] |
| **4-8** | 1-((*β*-D-Glucopyranosyloxy)methyl)-5,6-dihydropyrrolizin-7-one | *L. cymbulifera* | root | [80] |
| **4-9** | 3,9-Dimethyl-5-nitropyrido(3,2,1-ij)quinazoline-1,7-dione | *L. duciformis* | rhizome | [109] |
| **4-10** | 1-(4'-Methylpyridazin-5'-yl)butane-1,2,3,4-tetraol | *L. duciformis* | rhizome | [109] |
| **4-11** | 2,7-Bis(isopropylimino)-2*H*,7*H*-dicyclopentacyclooctene-4,9-diol | *L. duciformis* | rhizome | [109] |
| **4-12** | N,N-Di(1-iminopropyl)propionamidine | *L. duciformis* | rhizome | [109] |
| | ***Flavonoids*** | | | |
| **4-13** | 6-Acetyl-8-methoxy-2,3-dimethylchromen-4-one | *L. macrophylla* | root and rhizome | [89] |
| **4-14** | (2*S*)-3'-Hydroxy-5',7-dimethoxyflavanone | *L. macrophylla* | root and rhizome | [89] |
| | ***Lignans*** | | | |
| **4-15** | 9*α*-Angeloyloxypinoresinol | *L. kanaitizensis* | root and rhizome | [110] |
| **4-16** | 4-((*β*-D-Glucopyranosyl)oxy)pinoresinol | *L. virgaurea* spp. *oligocephala* | whole plant | [66] |
| **4-17** | Narynenol | *L. narynensis* | root | [111] |
| | ***Others*** | | | |
| **4-18** | 2-Isoprepenyl-6-acetyl-8-methoxy-1,3-benzodioxin-4-one | *L. intermedia* | root | [112] |
| **4-19** | 6-Acetyl-7-hydroxy-2-isopropylidene-benzo(1,4)dioxin-3-one | *L. stenocephala* | root | [113] |
| **4-20** | 2,5-Dihydroxy-6,7-dimethylnaphthoquinone | *L. vellerea* | whole plant | [6] |
| **4-21** | Ligumedial | *L. intermedia* | root and rhizome | [114] |
| **4-22** | Ligumediaoic acid | *L. intermedia* | root and rhizome | [114] |
| **4-23** | 7,8-Dimethoxy-1,4-dimethyldibenzofuran | *L. stenocephala* | root and leaves | [105] |
| **4-24** | 1,2,4-Trimethyl-7,8-dimethoxy-dibenzofuran | *L. caloxantha* | root | [115] |
| **4-25** | 4-*O*-(6-Hydroxy-7(9)-dehydro-6,7-dihydrogeranyl)-coniferyl alcohol | *L. duciformis* | root | [116] |
| **4-26** | 4-*O*-(7-Hydroxy-5,6*E*-dehydro-6,7-dihydrogeranyl)-coniferyl alcohol | *L. duciformis* | root | [116] |
| **4-27** | 4-*O*-(6-Hydroperoxy-7(9)-dehydro-6,7-dihydrogeranyl)-coniferyl alcohol | *L. duciformis* | root | [116] |
| **4-28** | 4-*O*-(7-Hydroperoxy-5,6*E*-dehydro-6,7-dihydrogeranyl)-coniferyl alcohol | *L. duciformis* | root | [116] |
| **4-29** | 4-*O*-(6-Hydroxy-7(9)-dehydro-6,7-dihydrogeranyl)-sinapyl alcohol | *L. duciformis* | root | [116] |
| **4-30** | 4-*O*-(6-Hydroxy-7(9)-dehydro-6,7-dihydrogeranyl)-coniferyl alcohol | *L. duciformis* | root | [116] |
| **4-31** | (*E*,*E*)-4-(7-Hydroperoxy-3,7-dimethylocta-2,5-dienyloxy)-syringenin | *L. intermedia* | root | [112] |
| **4-32** | (*E*)-4-(6-Hydroperoxy-3,7-dimethylocta-2,7-dienyloxy)-syringenin | *L. intermedia* | root | [112] |
| **4-33** | No name | *L. nelumbifolia* | root | [117] |
| **4-34** | No name | *L. nelumbifolia* | root | [117] |
| **4-35** | No name | *L. nelumbifolia* | root | [117] |
| **4-36** | 3,4,5-Trimethoxycinnamyl angelic acid ester | *L. veitchiana* | root | [4] |
| **4-37** | 4-((3',4'-Dihydroxycinnamoyl)-oxy)-methyl cinnamate | *L. vellerea* | whole plant | [6] |
| **4-38** | 2-Acetyl-5,6-dimethoxybenzofuran | *L. przewalskii* | root | [55] |
| **4-39** | 2-Propenyl-5-acetyl-7-hydroxy-2,3-dihydrobenzofuran | *L. przewalskii* | root | [55] |
| **4-40** | 5-Acetyl-7-methoxybenzofuran | *L. przewalskii* | root | [55] |
| **4-41** | No name | *L. nelumbifolia* | root | [117] |
| **4-42** | 6-Hydroxy-3*α*-methoxytremetone | *L. stenocephala* | root and leaves | [105] |
| **4-43** | 2-Acetyl-5-isopentenyl-6-methylbenzofuran | *L. veitchiana* | root | [4] |
| **4-44** | 2,2'-(1",1"-Dimethyl-3"-methoxy-3"-methyl-1",3"-propanediyl)bis (5,6-dimethoxybenzofuran) | *L. stenocephala* | root and leaves | [105] |
| **4-45** | Stenocephalin A | *L. stenocephala* | root | [118] |
| **4-46** | Ligustenin C | *L. stenocephala* | root | [119] |
| **4-47** | Ligustenin B | *L. stenocephala* | root | [119] |
| **4-48** | Ligustenin D | *L. stenocephala* | root | [119] |
| **4-49** | (+)-Ligulacephalin A | *L. stenocephala* | root | [120] |
| **4-50** | (–)-Ligulacephalin A | *L. stenocephala* | root | [120] |
| **4-51** | (+)-Ligulacephalin B | *L. stenocephala* | root | [120] |
| **4-52** | (–)-Ligulacephalin B | *L. stenocephala* | root | [120] |

| | | | | |
|---|---|---|---|---|
| **4-53** | (+)-Ligulacephalin C | *L. stenocephala* | root | [120] |
| **4-54** | (–)-Ligulacephalin C | *L. stenocephala* | root | [120] |
| **4-55** | Stenocephalin B | *L. stenocephala* | root | [118] |
| **4-56** | Stenocephalin C | *L. stenocephala* | root | [118] |
| **4-57** | (*R*)-(+)-Ligulaodonin A | *L. odontomanes* | whole plant | [101] |
| **4-58** | Ligustenin A | *L. stenocephala* | root | [119] |
| **4-59** | (2*α*,3*β*,5*α*)-2-(Acetyloxy)-9-methoxy-5-(methoxycarbonyl)-2,3-dimethylheptano-5-lactone | *L. dentata* | root | [83] |
| **4-60** | (2*β*,4*β*)-2-Ethyl-5-hydroxy-5-(methoxy-carbonyl)-4,5-dimethylpentano-4-lactone | *L. dentata* | root | [83] |
| **4-61** | Euparin | *L. caloxantha* | root and rhizome | [121] |
| **4-62** | 6-Methoxy–euparin | *L. caloxantha* | root and rhizome | [121] |
| **4-63** | 3,4-Dicaffeoylquinic acid | *L. fischeri* var. *spiciformis* and *L. stenocephala* | whole plant and leaves | [122] [123] |
| **4-64** | 3,5-Dicaffeoylquinic acid | *L. stenocephala* | leaves | [123] |

## 2 Biological Activities

**2.1 Antibacterial Activity:** In 2003, Li *et al* reported that eremophilane sesquiterpenoid lactones **1-20** and **1-51**, isolated from *L. sagitta*, showed antibacterial activity against *Staphylococcus aureus*, *Bacillus subtilis* and *Escherichia coli* according to the paper-disk method[26]. In 2009, another eremophilane sesquiterpenoid lactone **1-41**, from *L. hodgsonii*, was reported showing weak antibacterial activity against *Bacillus subtilis* with the MIC of 128 μg/mL[37].

**2.2 Cytotoxic Activity:** In 2006, Wu *et al* reported the cytotoxic activities of eremoligularin (**1-130**) and bieremoligularolide (**1-268**). The result revealed that bieremoligularolide (**1-268**) showed strong cytotoxicities: $IC_{50}$ = 5.5, 16.1, and 8.9 μM against HL-60, SMMC-7721, and HeLa cells, respectively. However, eremoligularin (**1-130**) showed no cytotoxicity against the above three cells ($IC_{50}$ > 100 μM)[15]. In addition, in 2008, an eremophilane sesquiterpenoid **1-152**, obtained from *L. veitchiana*, was reported to exhibited significant inhibiting activities on the growth of lung-cancer (A549) and stomachcancer (BCG823) cell lines, with $IC_{50}$ values of 10.27 (A549) and 31.14 (BCG823) μg/mL, respectively[67]. While in 2010, an bisabolane sesquiterpene **1-241** was found showing significant cytotoxicity against human lung carcinoma (A-549), human breast adenocarcinoma (MCF-7), epidermoid carcinoma of the nasopharynx (KB), and vincristine-resistant nasopharyngeal (KBVIN) cell lines, with $EC_{50}$ values of 3.4 (A549), 0.8 (MCF-7), 1.0 (KB), and 0.9 (KBVIN) μg/mL, respectively[79].

**2.3 Protein Tyrosine Phosphatase Inhibitory Activity:** In 2009, an eremophilane lactone **1-43**, from the roots of *L. fischeri*, was evaluated for the inhibitory activity against protein tyrosine phosphatase (PTP1B) *in vivo* by Deng *et al*[23]. The experiment data indicated moderate inhibitory activity with $IC_{50}$ = 1.34 μM.

**2.4 Insecticidal and Antifeedant Activities:** The plant *L. caloxantha* has been used as a folk medicine in the Naxi nationality in Yunnan province for years. In 2005, a phytochemical investigeation on the roots and rhizomes of this plant by Li *et al* led to the isolation of two benzofuran compounds, euparin (**4-61**) and 6-methoxy-euparin (**4-62**)[121]. The bioactivity assay revealed that both of the two compounds showed significant insecticidal and antifeedant activities. This conclusion may provide an explanation why *L. caloxantha* is used as a folk anti-insect agent.

**2.5 Antihepatotoxicity and Antioxidative Activity:** It has been reported that the MeOH extract (LFS) of *L. fischeri* var. *spiciformis* and its active component, 3,4-dicaffeoylquinic acid (DCQA) (**4-63**), showed significant antihepatotoxicity, the action mechanism of which was investigated by Choi *et al* in 2004[122]. The result showed that both LFS and DCQA resultantly prevented hepatotoxicity via antioxidative mechanism. Thus, it was proposed that antihepatotoxicity of LFS was based on the antioxidative action of DCQA.

**2.6 Antithrombotic and Anticoagulating Activity:** In 2008, Yoon *et al* reported that the leaf extract of *L. stenocephala* showed the highest anti-platelet aggregating activity. The active fraction inhibited the platelet aggregation up to above 80% and its blood coagulating time also showed similar effect to aspirin (0.2 μg/mL), known as an anti-thrombus compound. An activity-guided seperation resulted in two antithrombus active compounds as 3,4-dicaffeoylquinic acid (**4-63**) and 3,5-dicaffeoylquinic acid (**4-64**). A further assay showed that the two active compounds has not only antiplatelet aggregating activity, but also has anticoagulating activity[123].

## 3 Conclusions

This review summarized the secondary metabolites reported from *Ligularia* species as well as their biological activities in recent decades. These conclusions indicate that *Ligularia* species may be a rich source of natural products with chemical and biological diversity.

### Acknowledgments

This work was supported by the Important Directional Project of the Chinese Academy of Sciences (No. KSCX2-EW-R-15) and the National Natural Science Foundation of China (No. 21075127).

**Open Access** This article is distributed under the terms of the Creative Commons Attribution License which permits any use, distribution, and reproduction in any medium, provided the original author(s) and source are credited.
